# A Comparison of Two Classes of Methods for Estimating False Discovery Rates in Microarray Studies

**DOI:** 10.6064/2012/519394

**Published:** 2012-07-08

**Authors:** Emily Hansen, Kathleen F. Kerr

**Affiliations:** ^1^Cancer Research and Biostatistics, Seattle, WA 98101, USA; ^2^Department of Biostatistics, University of Washington, Seattle, WA 98195, USA

## Abstract

The goal of many microarray studies is to identify genes that are differentially expressed between two classes or populations. Many data analysts choose to estimate the false discovery rate (FDR) associated with the list of genes declared differentially expressed. Estimating an FDR largely reduces to estimating *π*_1_, the proportion of differentially expressed genes among all analyzed genes. Estimating *π*_1_ is usually done through *P*-values, but computing *P*-values can be viewed as a nuisance and potentially problematic step. We evaluated methods for estimating *π*_1_ directly from test statistics, circumventing the need to compute *P*-values. We adapted existing methodology for estimating *π*_1_ from *t*- and *z*-statistics so that *π*_1_ could be estimated from other statistics. We compared the quality of these estimates to estimates generated by two established methods for estimating *π*_1_ from *P*-values. Overall, methods varied widely in bias and variability. The least biased and least variable estimates of *π*_1_, the proportion of differentially expressed genes, were produced by applying the “convest” mixture model method to *P*-values computed from a pooled permutation null distribution. Estimates computed directly from test statistics rather than *P*-values did not reliably perform well.

## 1. Introduction

 Gene expression microarrays are a standard tool for large-scale measurement of gene expression. Microarrays are widely used to detect genes that are differentially expressed (DE) across different groups. Methodology for detecting DE genes has matured over the past decade. Methods have evolved from simple fold-change rules, to the use of classical statistical methods, to the use of test statistics developed specifically for the microarray context (here termed Specialized Test Statistics).

 The search for DE genes is usually done in the framework of statistical hypothesis testing. A hypothesis test is performed for each gene. Since microarray studies usually involve tens of thousands of genes, detecting DE genes automatically involves multiple testing issues. Rather than controlling false positives through the traditional family-wise error rate (FWER), most researchers prefer to consider the false discovery rate (FDR). The false discovery rate is an alternative to FWER that was originally proposed by Benjamini and Hochberg [1]. The FDR is the expected proportion of false positives among all the genes declared DE. For example, a FDR of 5% means that among all genes declared DE, 5% of these are truly non-DE on average.

Controlling the FWER is too conservative in the microarray setting, because usually investigators are willing to get a small proportion of false positives in exchange for a sizeable list of potentially DE genes for further study. There is a near-consensus that FDR-estimation procedures are the preferred method for addressing multiple testing in the microarray context [2, 3]. A typical procedure is as follows. (1) Compute a test statistic for every gene. (2) Obtain a *P*-value for every gene. (3) For some threshold *α* close to 0 (e.g., *α* = 0.001), call all genes with *P*-values less than *α* significant. (4) Estimate the FDR associated with the list of significant genes. In reality steps (3) and (4) might be done iteratively, with the *P*-value threshold adjusted depending on the estimated FDR resulting from step (4). Next, we review methodology for these 4 steps, which will introduce the question this paper will address.

 Step (1) involves the choice of test statistic. The earliest approaches to identifying DE genes were simple fold-change rules. An example of a fold-change rule is to declare a gene DE if its average expression level varies by more than a factor of two between the comparison groups. However, such rules are generally considered unsatisfactory because they do not incorporate the variability of the data, and there is no associated level of confidence in the conclusion of declaring a gene DE [2]. It was natural for statisticians to propose classical test procedures instead of fold-change rules. Let *X*_*jk*_ be the (possibly log-transformed) level of expression of a given gene in sample *j* of population *k*. Let *n*_*k*_ be the number of samples drawn from population *k*. Define $\overset{-}{X_{k}}=\left(1/n_{k}\right)\sum_{j=1}^{n_{k}}X_{jk}$ to be the sample average level of expression of the gene in population *k*. Also define $s_{k}^{2}=\left(1/\left(n_{k}-1\right)\right)\sum_{j=1}^{n_{k}}{\left(X_{jk}-\overset{-}{X_{k}}\right)}^{2}$ to be the sample variance of the expression level of the gene in population *k*. Typically expression levels are compared across two populations or groups (*k* = 1,2). To identify DE genes, one could use the classical two-sample *t-*statistic:

(1) $$\begin{array}{c} t=\frac{{\overset{-}{X}}_{1}-{\overset{-}{X}}_{2}}{\sqrt{\left(s_{1}^{2}/n_{1}\right)+\left(s_{2}^{2}/n_{2}\right)}}. \end{array}$$

However, because of the large number of genes and the nature of microarray data, this statistic is not well suited for discriminating DE genes. The denominator of the *t-*statistic relies on estimates of the expression variances. With the sample sizes typical of most microarray studies (only a few samples per group), these estimates are very unstable. Given the large number of genes in microarray studies, some genes will exhibit a low variance by chance. In real data one often finds that the genes with the largest *t-*statistics are those with the smallest denominators, not necessarily those that are DE between groups.

Tusher et al. [4] proposed a specialized statistic for microarrays, known as the “SAM” or *s-*statistic,

(2) $$\begin{array}{c} s=\frac{{\overset{-}{X}}_{1}-{\overset{-}{X}}_{2}}{\delta +\sqrt{\left(s_{1}^{2}/n_{1}\right)+\left(s_{2}^{2}/n_{2}\right)}}. \end{array}$$

Note that the *s-*statistic is identical to the *t-*statistic with the addition of a constant, *δ*, to the denominator. The constant *δ* has the effect of stabilizing the denominator of the *t-*statistic. We will refer to *δ* as the stabilizing constant.

 There is no consensus on the best way to choose *δ*. Tusher et al. [4] state that they choose *δ* to ensure that the variance of the *s-*statistic “is independent of gene expression.” Broberg [5] used the 5th percentile of all *t-*statistic denominators as *δ*, and Xie et al. [6] used the median. Efron et al. [7] compared 5 choices of *δ*: 0; 5th, 50th, and 90th percentiles, and the limit as it approached infinity and found the 90th percentile worked best.

In addition to the SAM statistic, other Specialized Test Statistics for microarrays have been developed. Specialized Test Statistics have overwhelmingly been found to outperform the *t-*statistic for detecting DE genes. In each of the papers mentioned above ([8–13]), simulations were included comparing the performance of the proposed Specialized Test Statistic to the classical *t-*statistic. Using real microarray data from a set of “spike-in” assays, Qin et al. [14] assessed the performance of six different statistics. Results unambiguously demonstrated superior performance of Specialized Test Statistics over the mean or *t-*statistic for identifying DE genes although there was no “clear winner” among the Specialized Test Statistics. Similarly, Zhang and Cao [15] employed both simulation and real “spike-in” data and demonstrated that Specialized Test Statistics performed comparably and clearly outperformed classical statistics like the *t-*statistic.

 Step (4) involves estimating the FDR associated with a list of genes declared significant. Many different “Mixture Model Methods” (MMMs) [16] have been developed to estimate the FDR for a list of genes declared DE. MMMs assume that there is a valid *P*-value computed for each gene to test the null hypothesis that the gene is not DE. MMMs consider these *P*-values as a mixture of *P*-values for genes for which the null hypothesis is true and *P*-values for which the null hypothesis is false. Estimators of the FDR have the form

(3) $$\begin{array}{c} \hat{\text{FDR}\left(\alpha \right)}=\;\frac{\alpha \hat{\pi_{0}}}{\hat{F_{p}}\left(\alpha \right)}=\;\frac{\alpha \left(1-\hat{\pi_{1}}\right)}{\hat{F_{p}}\left(\alpha \right)}, \end{array}$$

where *α* is the *P*-value threshold, *π*_0_ is the proportion of non-DE genes, *π*_1_ = (1 − *π*_0_) is the proportion of DE genes, and *F*_*p*_(·) is the cumulative distribution function of all *P*-values. Most MMMs use the observed number of *P*-values less than *α* to estimate *F*_*p*_(*α*). Therefore, most MMMs differ only in their estimates of *π*_1_.

 In order to compute a *P*-value—step (2)—one needs to know the distribution of the test statistic under the null hypothesis. Obtaining an empirical null distribution by permutation is a very popular choice in the microarray context. However, Kerr [17] showed that *P*-values resulting from permutation tests and MMMs may be incompatible since permutation-test *P*-values may not satisfy all of the assumptions implicit in MMM methodology. Therefore, estimating the FDR directly from test statistics—skipping step (2) altogether—could be advantageous.

There are few tools available to estimate the FDR directly from test statistics. One such tool, “locfdr” [18], operates directly on test statistics, but has been found to be highly sensitive to minor changes or transformations of the test statistics [17]. Therefore, in this paper, we investigate whether the methodology of “fdrtool” [19, 20] can be easily adapted to estimate FDRs directly from Specialized Test Statistics.

There are many different Specialized Test Statistics to choose from; we use the SAM-statistic in our investigation due to its simplicity and popularity. We compare results to a procedure in current common practice, which is to use a variant of a permutation-test *P*-value together with a MMM for *P*-values. In this approach, a single null distribution of the test statistic is estimated by pooling all the permutation null test statistics across genes. Kerr [17] showed that such “pooled null *P*-values” are different from permutation test *P*-values. However, “pooled null *P*-values” have the attractive feature that they have a monotone relationship with the test statistic. For our second class of methods for estimating *π*_1_, we computed pooled null permutation *P*-values and then estimated the FDR with an MMM. We used two MMMs that we have seen to work well: “*q*value” [21] and “convest” [22].

## 2. Results and Discussion

### 2.1. Methodology

The methodology of “fdrtool” is not designed to take *s-*statistics or any other Specialized Test Statistics as input. Since “fdrtool” accepts *t-*statistics, and *s-*statistics are similar to *t-*statistics, we investigated whether we could apply “fdrtool” to *s-*statistics and get accurate results. Our idea was to compute both *s-*statistics and *t-*statistics on the same data, and then rescale the *s-*statistics to have the same spread as the corresponding *t-*statistics. We considered two measures of variability: standard deviation and interquartile range. In addition, we considered four different *s-*statistics, each using a different stabilizing constant term in the denominator. We designed simulations to investigate the accuracy of using “fdrtool” to estimate FDR's from *s-*statistics in this way. Our simulated data were based on real microarray data.

 As a simple example, suppose that we have 100 genes with measured expression levels on two samples from two groups we wish to compare. We compute a *t-*statistic and an *s-*statistic for each gene. Due to the addition of the stabilizing constant in the denominator of the *s-*statistic, the 100 *s-*statistic values will be closer to zero than the 100 *t-*statistic values. In other words, the addition of *δ* to the denominator pulls the *s-*statistic values toward zero, so that the variance of the *s-*statistics across genes is smaller than the variance of the *t-*statistics.

Accordingly, we used a measure of the difference in variability of the statistics across genes as the rescaling factor to rescale the *s-*statistics. We examined two measures of variability: the standard deviation (SD) and the interquartile range (IQR). We took the ratio of the variability of the *t-*statistic to the variability of the *s-*statistic as our rescaling factor. For the SD, we transformed the *s-*statistics by multiplying them by the factor (SD_*T*_/SD_*S*_), where SD_T_ and SD_S_ are the standard deviations across genes of *t-*statistics and *s-*statistics, respectively. The rescaling factor for the IQR was similarly defined, with IQR in the place of SD. Note that rescaling the *s-*statistics maintains their rank order, which retains the *s-*statistic's advantages for accurately detecting DE genes.

In computing *s-*statistics one must choose the value of the stabilizing constant *δ* in the *s-*statistic denominator. We considered four choices for *δ*, and so defined four different *s-*statistics: the *s*30, *s*50, *s*70, and *s*90. The *s*30-statistic uses the 30th percentile of all *t-*statistic denominators as *δ*, the *s*50-statistic uses the 50th percentile of all *t-*statistic denominators as *δ*, and so forth. In summary, for each simulated data set, we computed the *t-*statistic and the four *s-*statistics. We transformed the *s-*statistics by two different rescaling factors (SD and IQR). When loading the rescaled *s-*statistics into “fdrtool,” we evaluated two different specifications to the software. We could specify that the input was *t-*statistics or *z-*statistics. We evaluated the performance of these five statistics using the four different combinations of rescaling factors and two different input options.

### 2.2. Design of Simulation Study

The design of the simulation study is that same as Kerr (2009) [17], and we describe it here briefly. We based simulations on real microarray data of EBV-transformed lymphoblastoid cell line tissue from 60 individuals with European ancestry (CEU) and 45 ethnic Chinese (CHB). There are data on 47,293 transcripts.

For each gene, we calculated the sample mean and sample standard deviation of that gene in each population group (CEU and CHB). We rounded the sample mean values to the nearest tenth digit, so that means could be unambiguously declared equal or unequal between groups. We simulated CEU and CHB sample data (where we knew the “truth” regarding the degree of differential expression) from independent normal distributions with parameters based on the values from the actual data. We simulated datasets of 10,000 transcripts.

We ran three types of simulations: EV, UV1, and UV2. In each simulation, a proportion of genes (*π*_1_) were differentially expressed in the mean. For the simulated DE genes, the difference in means for the simulated CEU and CHB samples was taken from the observed sample means in the real data. The variances of the simulated CEU and CHB samples differed between the three simulation types.

In the EV (“equal variance”) simulations, the standard deviation of both the simulated CEU sample and the simulated CHB sample came from the observed standard deviation from the CEU data. This was not the case in the UV (“unequal variance”) simulations. In the UV1 simulations, the standard deviation of the simulated CEU sample came from the observed standard deviation from the CEU data, and the standard deviation of the simulated CHB sample came from the observed standard deviation from the CHB data. In the UV2 simulations, this was reversed: the standard deviation of the simulated CEU sample came from the observed standard deviation from the CHB data, and the standard deviation of the simulated CHB sample came from the observed standard deviation from the CEU data.

 We initially simulated data for four different values of *π*_1_: 0.01, 0.05, 0.10, and 0.25, and three different sample sizes: large, intermediate, and small. Letting nCEU and nCHB denote the sample sizes for CEU and CHB, respectively, our sample sizes were as follows: large (nCEU, nCHB) = (60, 45), intermediate (nCEU, nCHB) = (16, 12), and small (nCEU, nCHB) = (8, 6). Note that all of the sample sizes maintain the 4 : 3 ratio of the original data. With three sample sizes, four values of  *π*_1_, three simulation types, two rescaling factors (SD and IQR), and two input specification options (*t-*score, *z-*score), there were a total of 144 different simulation scenarios. After examining the results, we performed additional EV simulations for *π*_1_ = 0.005, 0.02, 0.03, and 0.04 for the three sample sizes, two rescaling factors, and two input specification options, adding an additional 48 simulation scenarios, for a total of 192 simulation scenarios. We repeated each scenario 20 times.

### 2.3. Evaluation of Adapted fdrtool Method

**Figure 1:**
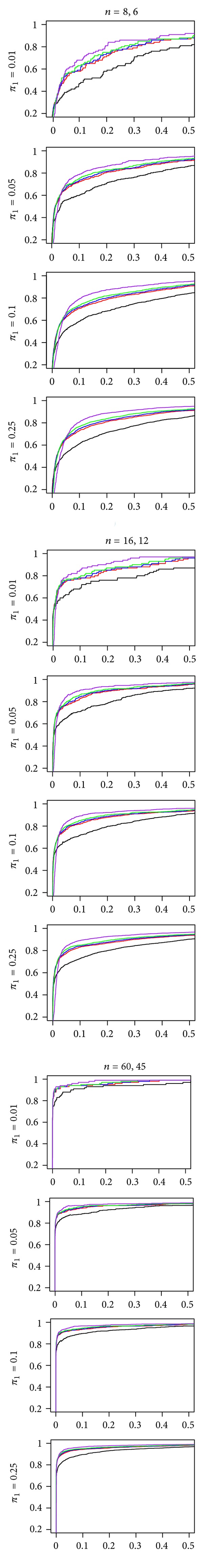
ROC curves for the *t-*statistic (black line), *s*30-statistic (red line), *s*50-statistic (blue line), *s*70-statistic (green line), and *s*90-statistic (purple line) in a type EV simulation. In each plot the horizontal axis is the false positive fraction and the vertical axis is the true positive fraction. The sample sizes in the two groups vary from 8 and 6 (left column) to 60 and 45 (right column). *π*_1_ denotes the proportion of differentially expressed genes.

First, we verified that the *s-*statistics outperformed the *t-*statistic in identifying DE genes.  Figure 1 shows that the *s-*statistics outperform the *t-*statistic across all sample sizes and values of *π*_1_. This difference in performance is more marked for the smaller sample sizes than the larger sample sizes, across all values of *π*_1_. The four *s-*statistics are generally close in performance. The *s*30-*, s*50-, and *s*70-statistics perform comparably. The *s*90-statistic (purple curve) stands slightly apart from the other three, giving less sensitivity at low false positive rates and better sensitivity at higher false positive rates. The difference in the ROC curves for the *s*90-statistic and the other *s-*statistics is more pronounced in the smaller sample sizes. However, the difference between the *s*90-statistic and the other *s-*statistics is not as large as the difference between the *t-*statistic and the four *s-*statistics.

**Figure 2:**
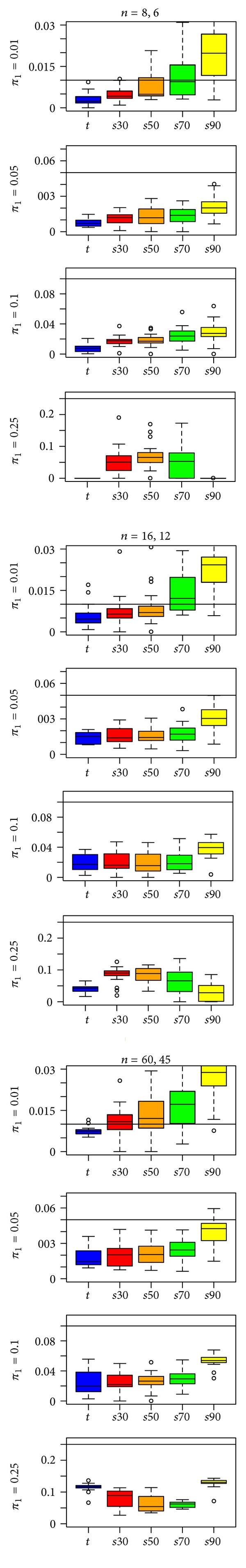
“fdrtool” estimates of *π*_1_ for the *t-*statistic and four *s-*statistics in a type EV simulation with the IQR rescaling factor and *t-*statistic input specification. A reference line is drawn at *π*_1_, the true proportion of differentially expressed genes.

We investigated the performance of “fdrtool” for *s-*statistics when “fdrtool” treated them as either *t-*statistics or *z-*statistics. Specifying in “fdrtool” that the inputted *s-*statistics were *t-*statistics (Figure 2) worked reasonably well for a low percentage of DE genes (*π*_1_ = 0.01, 0.05). In the first row of Figure 2, we see that the estimates of *π*_1_ have a modest conservative bias for the *s*30- and *s*50-statistics in the two smaller sample sizes. However, for higher proportions of DE genes (*π*_1_ = 0.1, 0.25), the “fdrtool” estimates of *π*_1_ are poor, with excessive conservative bias. This result held regardless of whether statistics were rescaled with IQR (Figure 1) or SD (see supplementary File 1 in supplementary material available online with doi:10.6064/2012/519394). However, SD-scaling tended to give less predictable results.

**Figure 3:**
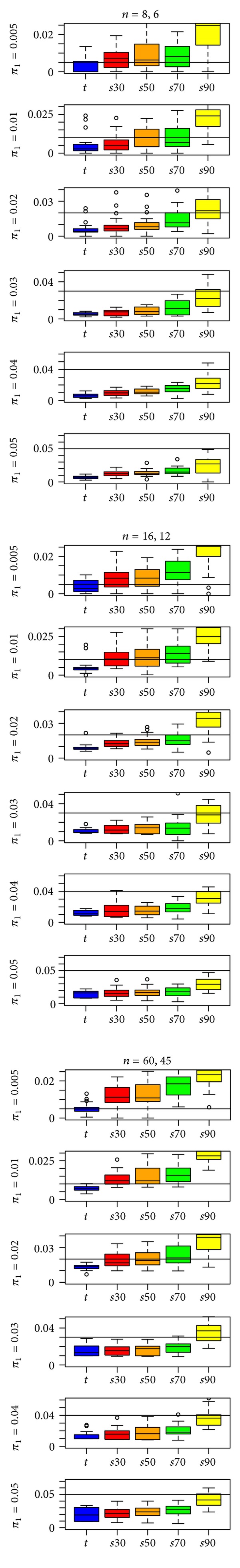
**“**fdrtool**”** estimates of *π*_1_ for the *t-*statistic and four *s-*statistics in a type EV simulation with the IQR rescaling factor and *t-*statistic input specification. A reference line is drawn at *π*_1_, the true proportion of differentially expressed genes.

Other aspects of the results presented in Figure 2 are notable. First, when *π* =0.25, there is a curvilinear trend in estimates of *π*_1_ as we move from the *t-*statistic at one extreme to the s90-statistic at the other. Second, the bias in the estimates of *π*_1_ when *t-*statistics are computed on the data tends to be at least as large as the bias for the *s-*statistics. This is surprising since “fdrtool” was developed for *t-*statistics. Third, there is a substantial difference between simulations with *π*_1_ = 0.01 and simulations with *π*_1_ = 0.05. When *π*_1_ = 0.01, estimates for the *s*70- and *s*90-statistics show anticonservative bias, whereas, when *π*_1_ = 0.05, these statistics show conservative bias.  Figure 3 expands upon Figure 2, with simulation results for *π*_1_ = 0.005, 0.01, 0.02, 0.03, 0.04, and 0.05. There is a decrease from anticonservative bias to conservative bias as *π*_1_ increases from 0.005 to 0.05.

**Figure 4:**
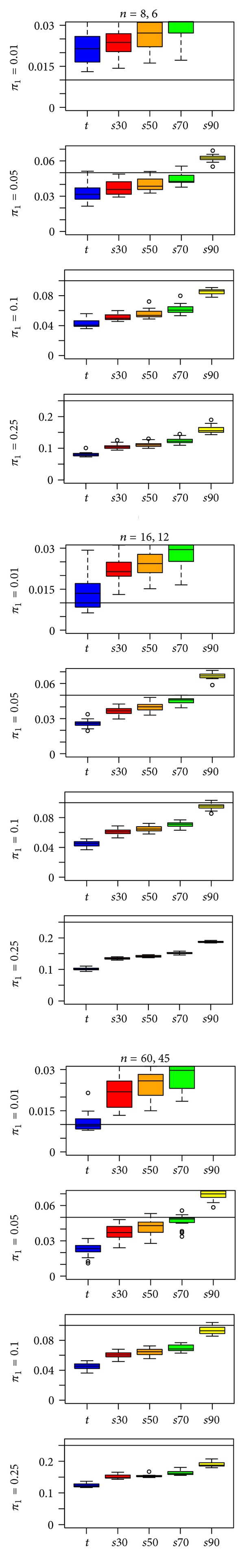
“fdrtool” estimates of *π*_1_ for the *t*-statistic and four s-statistics in a type EV simulation with the IQR-rescaling factor and *z-*statistic input specification. A reference line is drawn at *π*_1_, the true proportion of differentially expressed genes.

In contrast to the results for the *t-*statistic input specification, telling “fdrtool” the inputted statistics were *z-*statistics (Figure 4) worked better for high percentages of DE genes (*π*_1_ = 0.10, 0.25), but showed anticonservative bias for low percentages of DE genes (*π*_1_ = 0.01). Bias and variability is mostly improved over Figure 2 except for *π*_1_ = 0.01. All results presented here are for the EV simulations; results for the UV1 and UV2 simulations were similar (see supplementary File 1).

## 3. Evaluation of Mixture Model Methods on Pooled Permutation Null *P*-Values

A popular approach in practice is to compute a variant of a permutation-test *P*-value. In this approach, a single null distribution of the test statistic is estimated by pooling all the permutation null test statistics across genes. “Pooled null *P*-values” can be computed from a single empirical null distribution. We also obtained estimates of *π*_1_ using pooled null *P*-values and then estimating the FDR with an MMM. We obtained estimates of *π*_1_ using two MMMs that we have seen to work well: “*q*value” [21] and “convest” [22].

**Figure 5:**
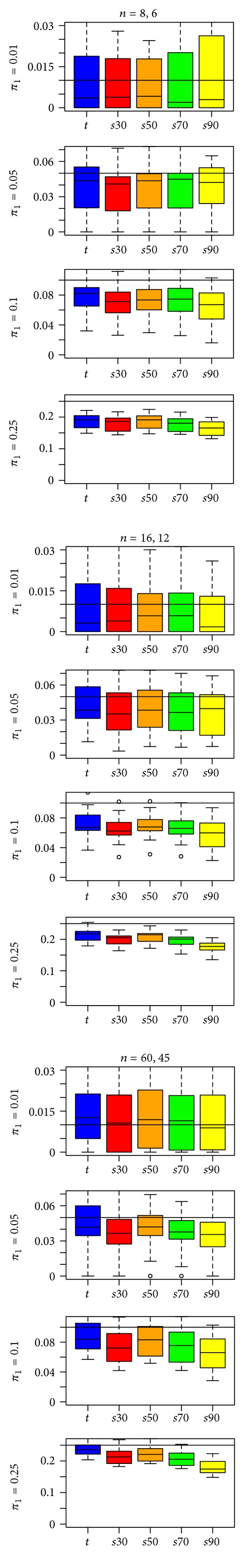
“*q*value” estimates of *π*_1_ for the *t-*statistic and four *s-*statistics in a type EV simulation. A reference line is drawn at *π*_1_, the true proportion of differentially expressed genes.

Figure 5 shows the “*q*value” results for the EV simulations. For a low percentage of DE genes (*π*_1_ = 0.01, 0.05), the “*q*value” results (top two rows of Figure 5) largely show less bias than the “fdrtool” results (top two rows of Figures 2 and 4). However, there are exceptions as, for example, the *s*50-and *s*70-statistics show less bias in the smaller sample sizes when *π*_1_ = 0.01 in the “fdrtool” results than in the “*q*value” results. The “*q*value” estimates show considerably greater variability than the “fdrtool” estimates, so it is not entirely clear that one method is better. For higher percentages of DE genes (*π*_1_ = 0.10, 0.25), the story is similar, with “*q*value” estimates generally showing less bias than “fdrtool” estimates, but greater variability. However, the higher variability in the “*q*value” results seems more clearly acceptable when *π*_1_ = 0.10, 0.25, given the dramatic reduction in bias. Interestingly, “*q*value” does not appear to perform worse for the *t-*statistic than it does for the *s-*statistics, whereas “fdrtool” almost always performs better for *s-*statistics. The comparison of the “fdrtool” results to the “*q*value” results was similar for the UV1 and UV2 simulations (see Additional File 1).

**Figure 6:**
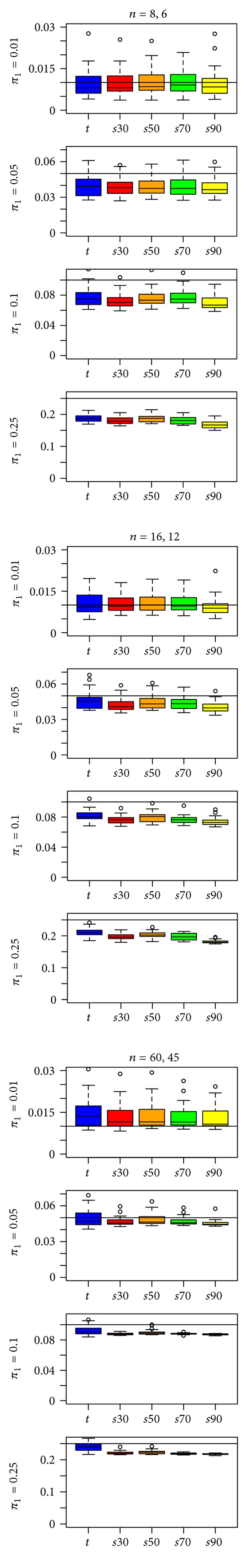
“convest” estimates of *π*_1_ for the *t-*statistic and four *s-*statistics in a type EV simulation. A reference line is drawn at *π*_1_, the true proportion of differentially expressed genes.

Figure 6 shows the “convest” estimates of *π*_1_ for the EV simulations. Overall, the “convest” estimates show less bias and comparable or less variability than the “fdrtool” or “*q*value” results. Thus, although it relies on *P*-values that could be considered invalid as permutation test *P*-values [17], “convest” might yield superior estimates of *π*_1_ (and hence FDR) in terms of bias and variability. Similar to the “*q*value” results, “convest” performs comparably for *t-*statistics as for *s-*statistics.

## 4. Discussion

The “convest” method took longer to compute than the “fdrtool” approach (approximately 20 times as long). However, computation time was on the order of 6.5 seconds for 1 set of 10,000 *P*-values and was not prohibitive. The more important difference is that “convest” requires *P*-values, which we computed via permutation, whereas the “fdrtool” is applied directly to test statistics and does not require any data permutations.

An important limitation of this study is that all simulations were based on one dataset. We also did not explore different correlation structures for the simulated gene expression.

## 5. Conclusions

We compared approaches for estimating *π*_1_, the proportion of differentially expressed genes, from microarray data. The approaches were in two classes: (1) adapting the methodology of “fdrtool” to Specialized Test Statistics and (2) applying mixture model methods (MMMs) to *P*-values computed from a pooled permutation null distribution. The best-performing method was in the second class, using the MMM “convest” to *P*-values computed from a pooled permutation null distribution. Overall, estimates of *π*_1_ exhibited the least bias and variability, and bias tended to be conservative rather than anti-conservative.

The first class of approaches for estimating *π*_1_ adapted existing methodology of “fdrtool” to *s-*statistics. Interestingly, “fdrtool” generally performed better for *s-*statistics than it did for *t-*statistics, even though the empirical modeling is designed for *t-*statistics. The performance of the “fdrtool”-based approach varied substantially on the proportion of differentially expressed gene. However, the “convest”-based approach outperformed the fdrtool approach in almost all scenarios.

## Supplementary Material

The Supplementary Material File contains the complete set of results for all simulation scenarios examined in the research.

## References

[1] Benjamini Y, Hochberg Y (1995). Controlling the false discovery rate: a practical and powerful approach to multiple testing. *Journal of the Royal Statistical Society Series B*.

[2] Allison DB, Cui X, Page GP, Sabripour M (2006). Microarray data analysis: from disarray to consolidation and consensus. *Nature Reviews Genetics*.

[3] Dupuy A, Simon RM (2007). Critical review of published microarray studies for cancer outcome and guidelines on statistical analysis and reporting. *Journal of the National Cancer Institute*.

[4] Tusher VG, Tibshirani R, Chu G (2001). Significance analysis of microarrays applied to the ionizing radiation response. *Proceedings of the National Academy of Sciences of the United States of America*.

[5] Broberg P (2003). Statistical methods for ranking differentially expressed genes. *Genome Biology*.

[6] Xie Y, Pan W, Khodursky AB (2005). A note on using permutation-based false discovery rate estimates to compare different analysis methods for microarray data. *Bioinformatics*.

[7] Efron B, Tibshirani R, Storey JD, Tusher V (2001). Empirical Bayes Analysis of a Microarray Experiment. *Journal of the American Statistical Association*.

[8] Baldi P, Long AD (2001). A Bayesian framework for the analysis of microarray expression data: regularized t-test and statistical inferences of gene changes. *Bioinformatics*.

[9] Lönnstedt I, Speed T (2002). Replicated microarray data. *Statistica Sinica*.

[10] Smyth GK (2004). Linear models and empirical bayes methods for assessing differential expression in microarray experiments. *Statistical Applications in Genetics and Molecular Biology*.

[11] Cui X, Hwang JT, Qiu J, Blades NJ, Churchill GA (2005). Improved statistical tests for differential gene expression by shrinking variance components estimates. *Biostatistics (Oxford, England)*.

[12] Kendziorski CM, Newton MA, Lan H, Gould MN (2003). On parametric empirical Bayes methods for comparing multiple groups using replicated gene expression profiles. *Statistics in Medicine*.

[13] Wright GW, Simon RM (2003). A random variance model for detection of differential gene expression in small microarray experiments. *Bioinformatics*.

[14] Qin L-X, Kerr KF, Boyles A (2004). Empirical evaluation of data transformations and ranking statistics for microarray analysis. *Nucleic Acids Research*.

[15] Zhang S, Cao J (2009). A close examination of double filtering with fold change and t test in microarray analysis. *BMC Bioinformatics*.

[16] Allison DB, Gadbury GL, Heo M (2002). A mixture model approach for the analysis of microarray gene expression data. *Computational Statistics and Data Analysis*.

[17] Kerr KF (2009). Comments on the analysis of unbalanced microarray data. *Bioinformatics*.

[18] Efron B (2007). Size, power and false discovery rates. *Annals of Statistics*.

[19] Strimmer K (2008). A unified approach to false discovery rate estimation. *BMC Bioinformatics*.

[20] Strimmer K (2008). fdrtool: a versatile R package for estimating local and tail area-based false discovery rates. *Bioinformatics*.

[21] Storey JD, Tibshirani R (2003). Statistical significance for genomewide studies. *Proceedings of the National Academy of Sciences of the United States of America*.

[22] Langaas M, Lindqvist BH, Ferkingstad E (2005). Estimating the proportion of true null hypotheses, with application to DNA microarray data. *Journal of the Royal Statistical Society Series B*.

