# Supplementary material for: A Comparison of Two Classes of Methods for Estimating False Discovery Rates in Microarray Studies
Source: Scientifica (Cairo). 2012 Jul 8;2012:519394. doi: 10.6064/2012/519394 (PMC3820438; doi:10.6064/2012/519394)
Supplement: Supplementary file 1 — The Supplementary Material File contains the complete set of results for all simulation scenarios examined in the research. [file 519394.f1.pdf]

APPENDIX A: RESULTS FOR EV SIMULATIONS

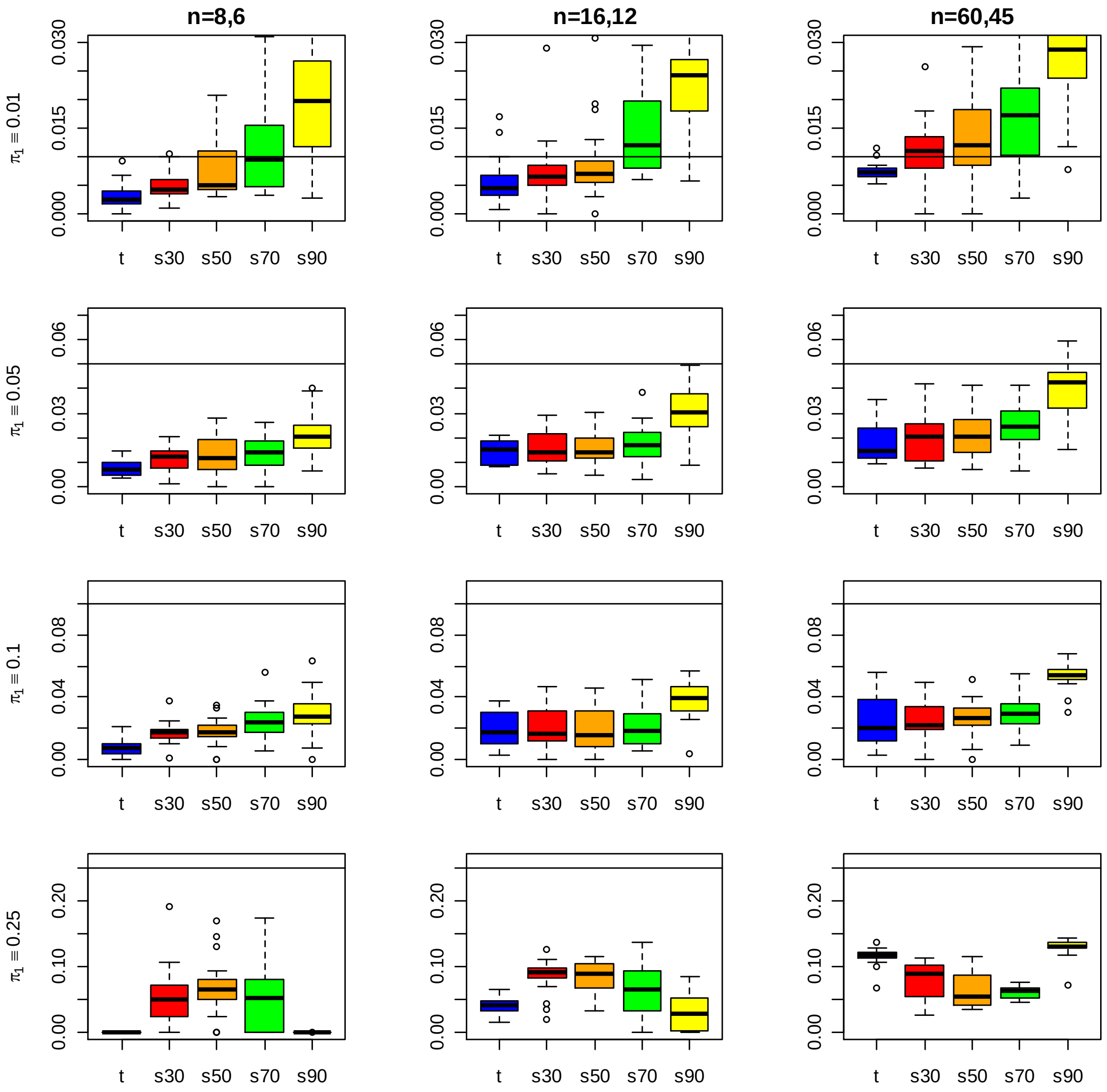

Figure 7. ‘fdrtool’ estimates of π_1 for the t-statistic and four s-statistics in a type EV simulation with the IQR rescaling factor and t-statistic input specification. A reference line is drawn at π_1, the true proportion of differentially expressed genes.

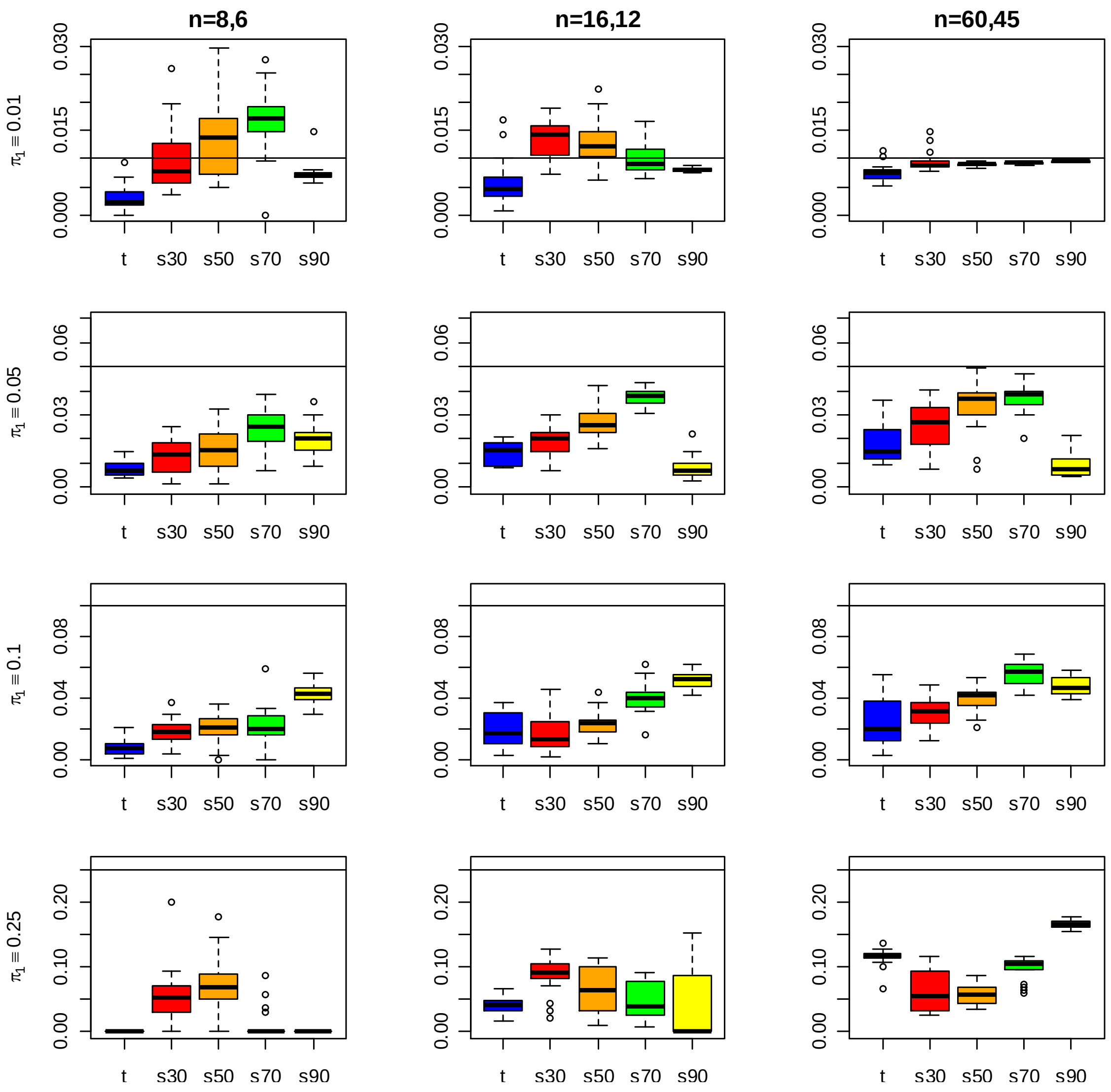

Figure 8. 'fdrtool' estimates of π_1 for the t-statistic and four s-statistics in a type EV simulation with the SD rescaling factor and t-statistic input specification. A reference line is drawn at π_1, the true proportion of differentially expressed genes.

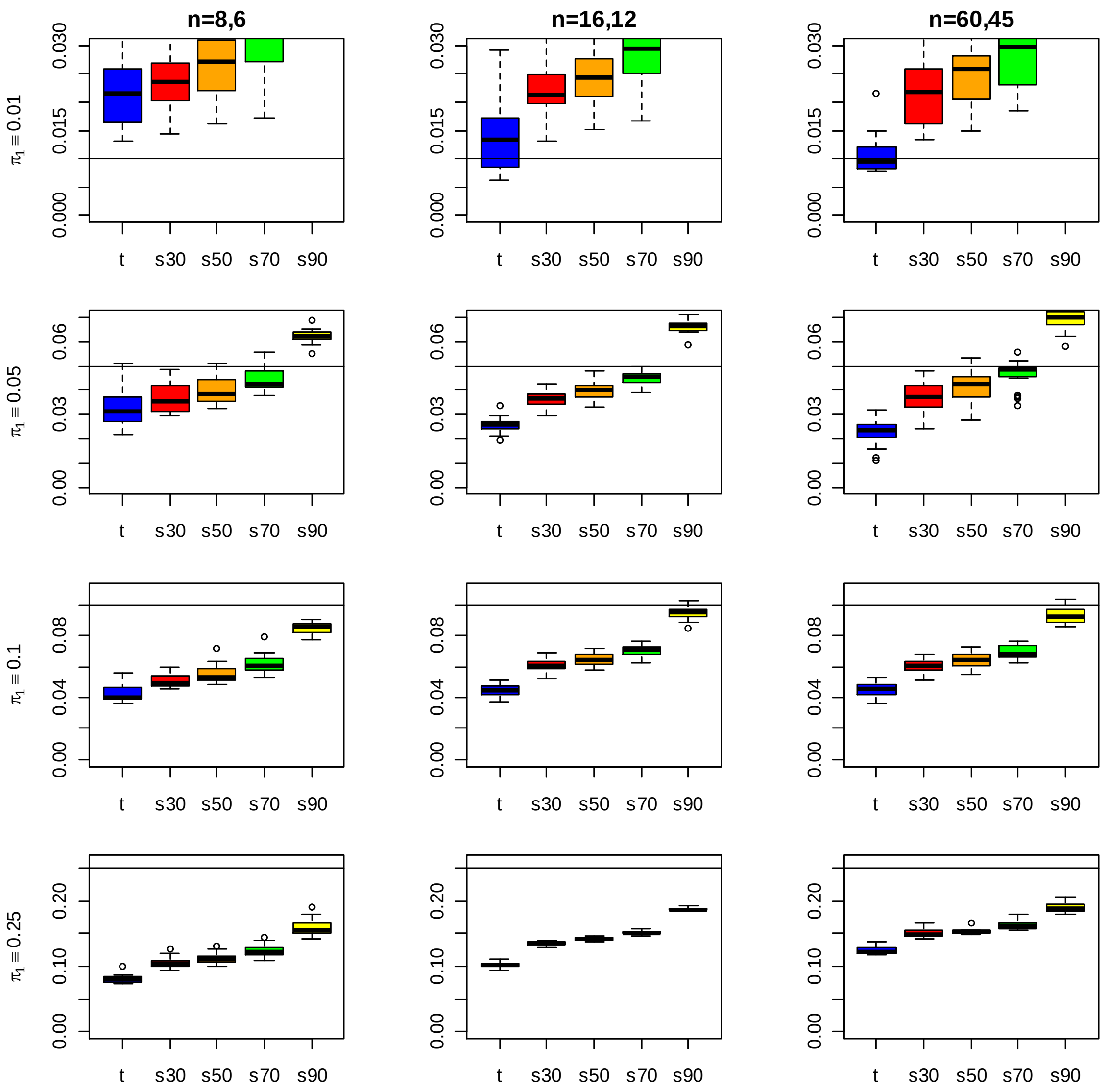

Figure 9. 'fdrtool' estimates of π_1 for the t-statistic and four s-statistics in a type EV simulation with the IQR rescaling factor and z-statistic input specification. A reference line is drawn at π_1, the true proportion of differentially expressed genes.

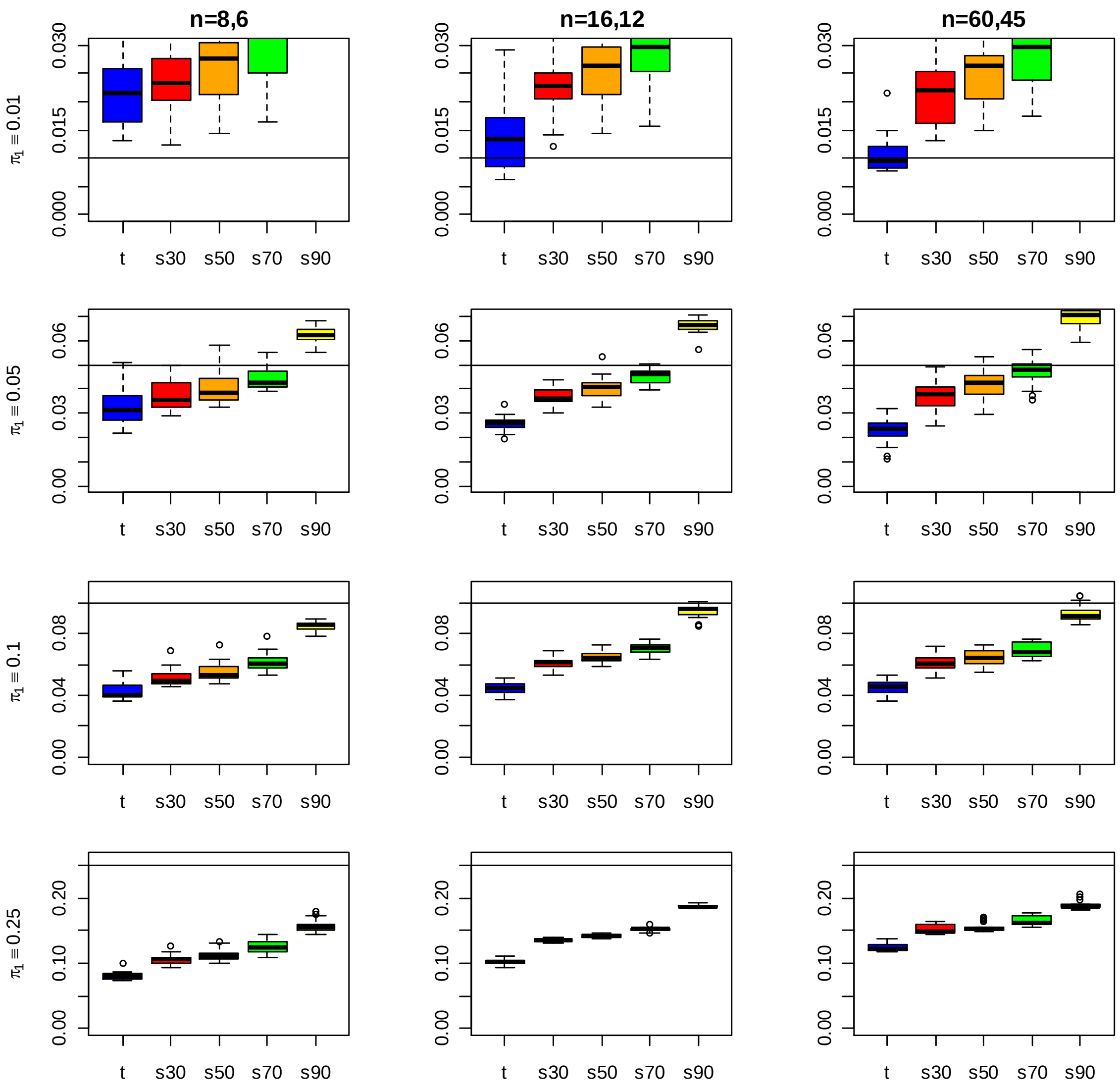

Figure 10. 'fdrtool' estimates of π_1 for the t-statistic and four s-statistics in a type EV simulation with the SD rescaling factor and z-statistic input specification. A reference line is drawn at π_1, the true proportion of differentially expressed genes.

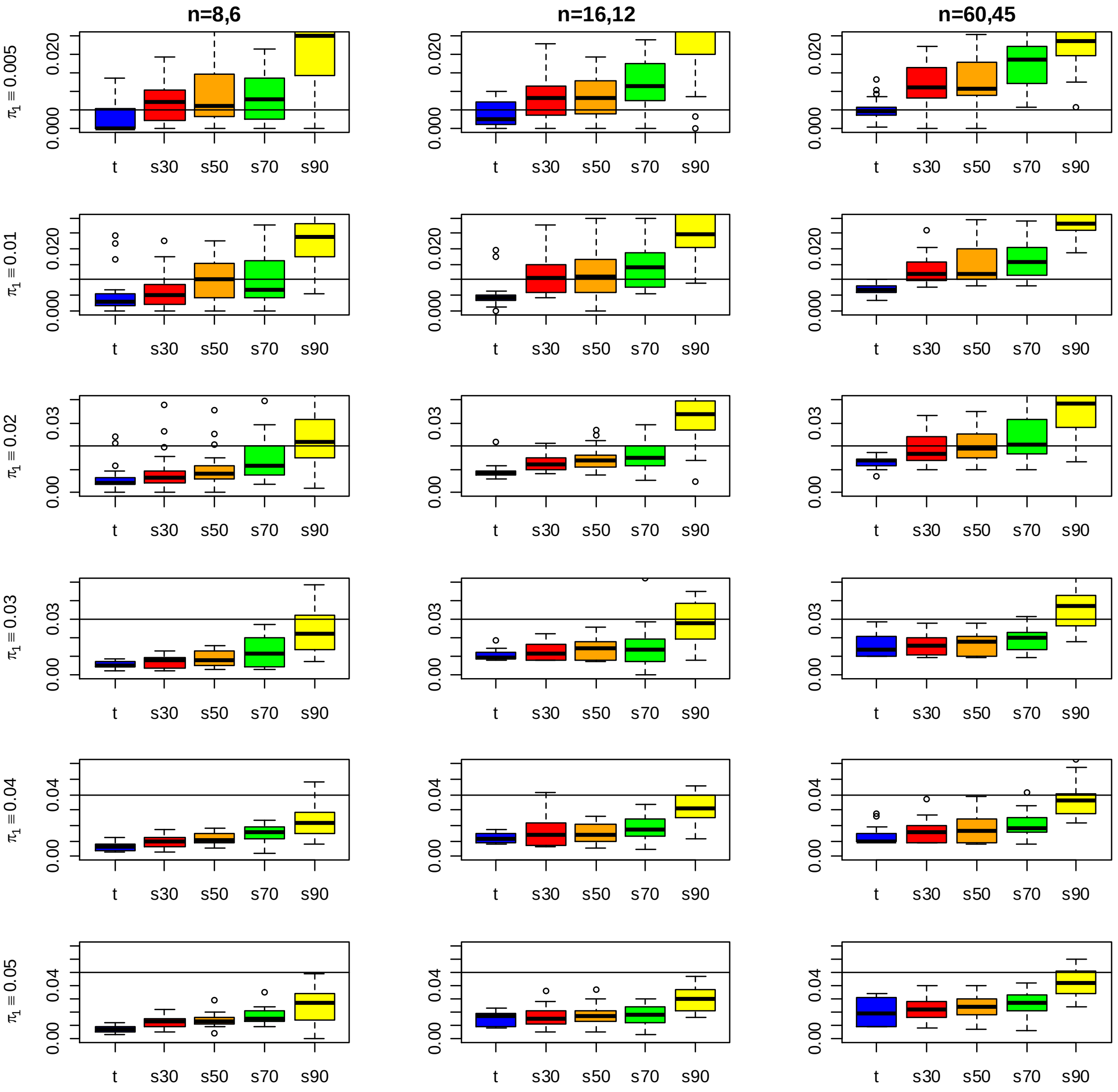

Figure 11. 'fdrtool' estimates of π_1 for the t-statistic and four s-statistics in a type EV simulation with the IQR rescaling factor and t-statistic input specification. A reference line is drawn at π_1, the true proportion of differentially expressed genes.

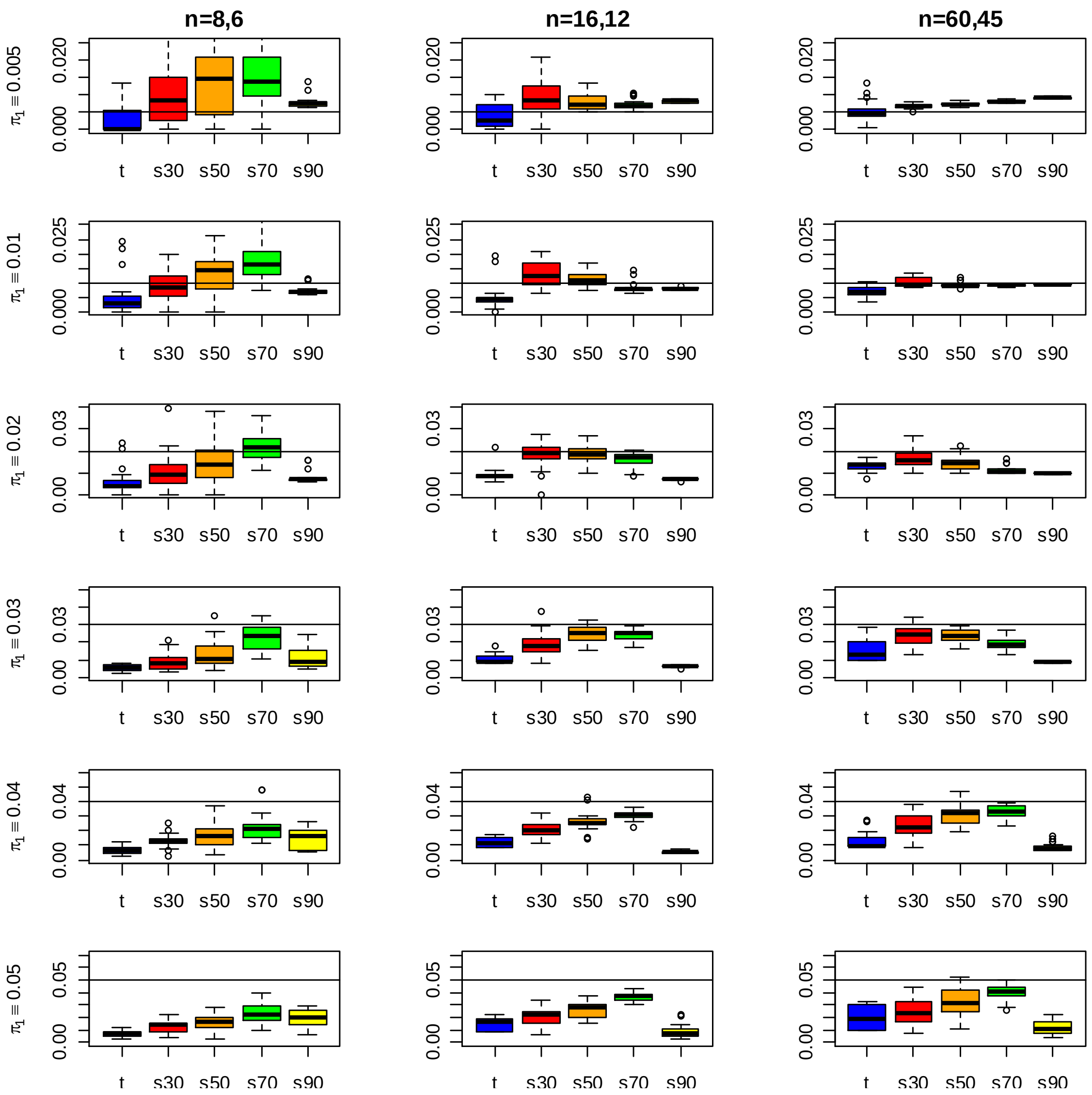

Figure 12. ‘fdrtool’ estimates of π_1 for the t-statistic and four s-statistics in a type EV simulation with the SD rescaling factor and t-statistic input specification. A reference line is drawn at π_1, the true proportion of differentially expressed genes.

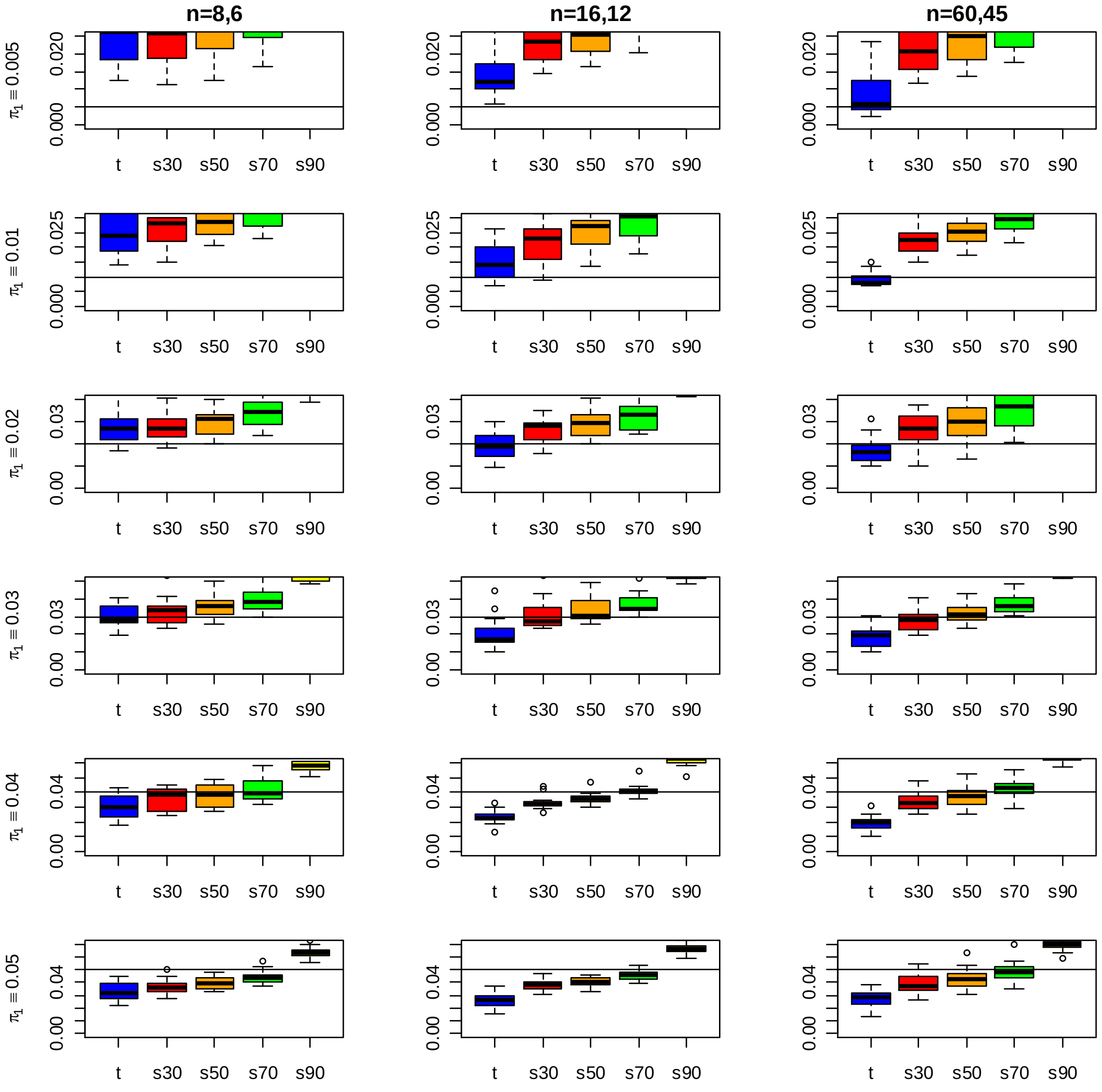

Figure 13. 'fdrtool' estimates of π_1 for the t-statistic and four s-statistics in a type EV simulation with the IQR rescaling factor and z-statistic input specification. A reference line is drawn at π_1, the true proportion of differentially expressed genes.

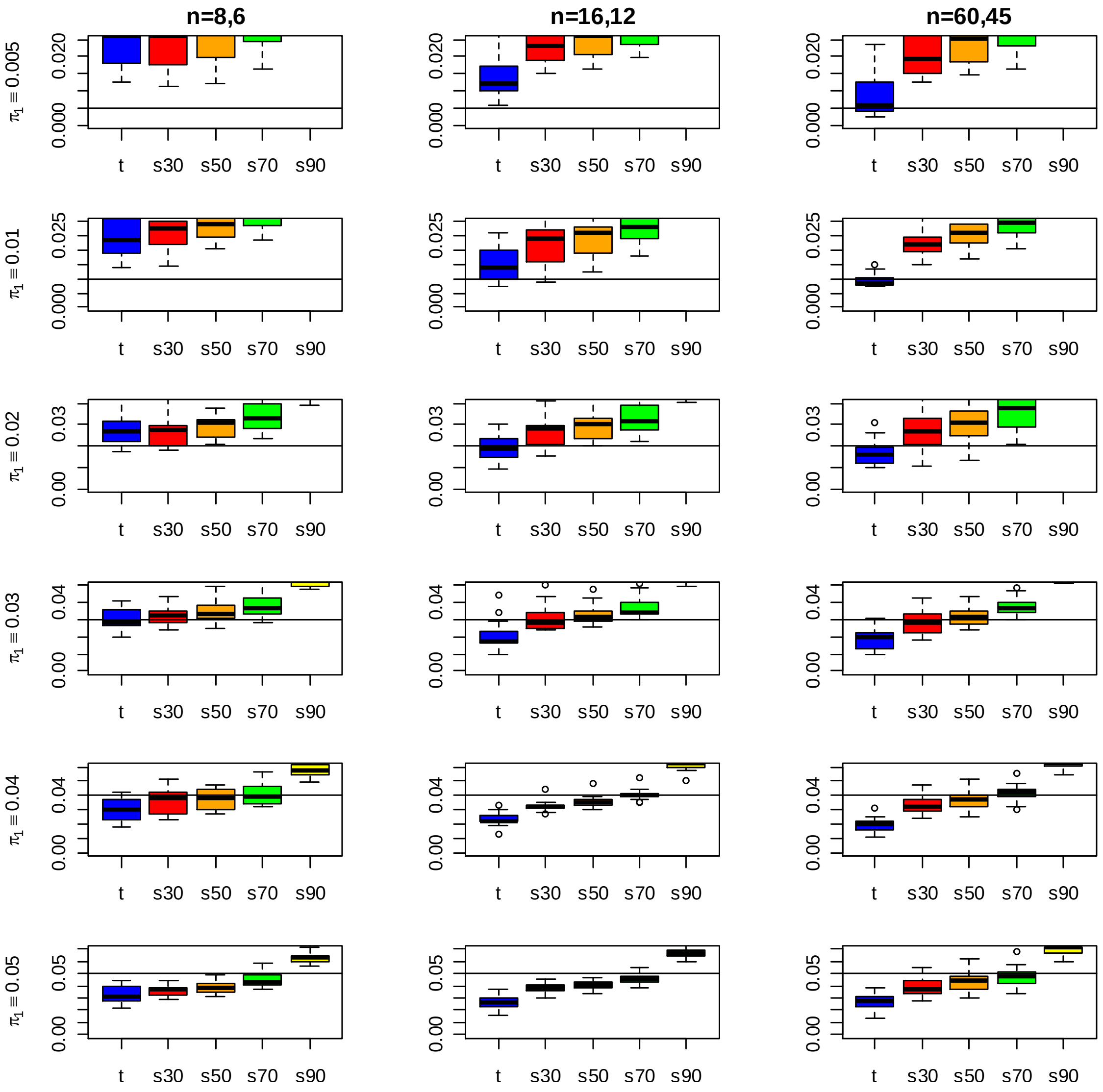

Figure 14. 'fdrtool' estimates of π_1 for the t-statistic and four s-statistics in a type EV simulation with the SD rescaling factor and z-statistic input specification. A reference line is drawn at π_1, the true proportion of differentially expressed genes.

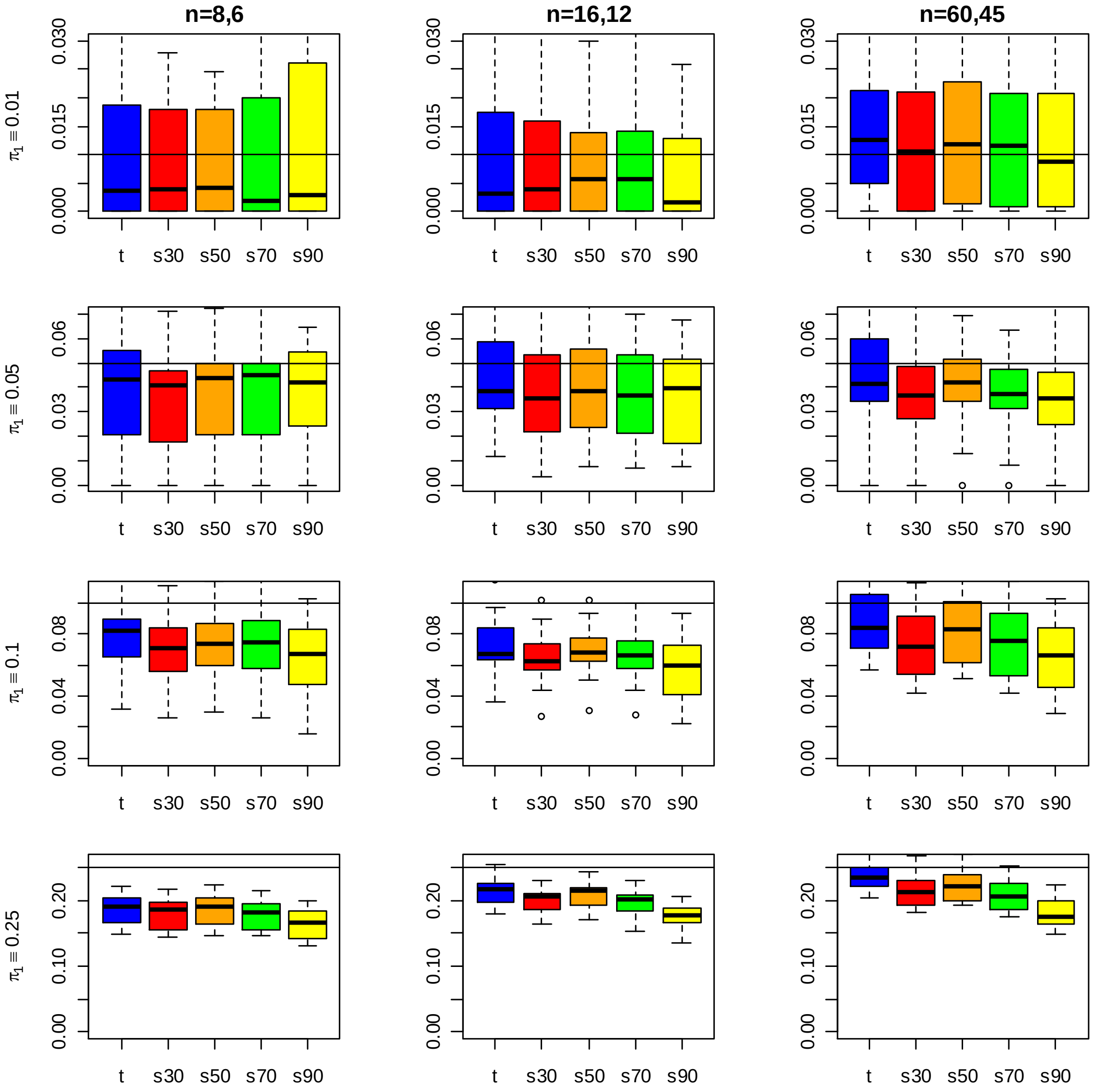

Figure 15. 'qvalue' estimates of π_1 for the t-statistic and four s-statistics in a type EV simulation. A reference line is drawn at π_1, the true proportion of differentially expressed genes.

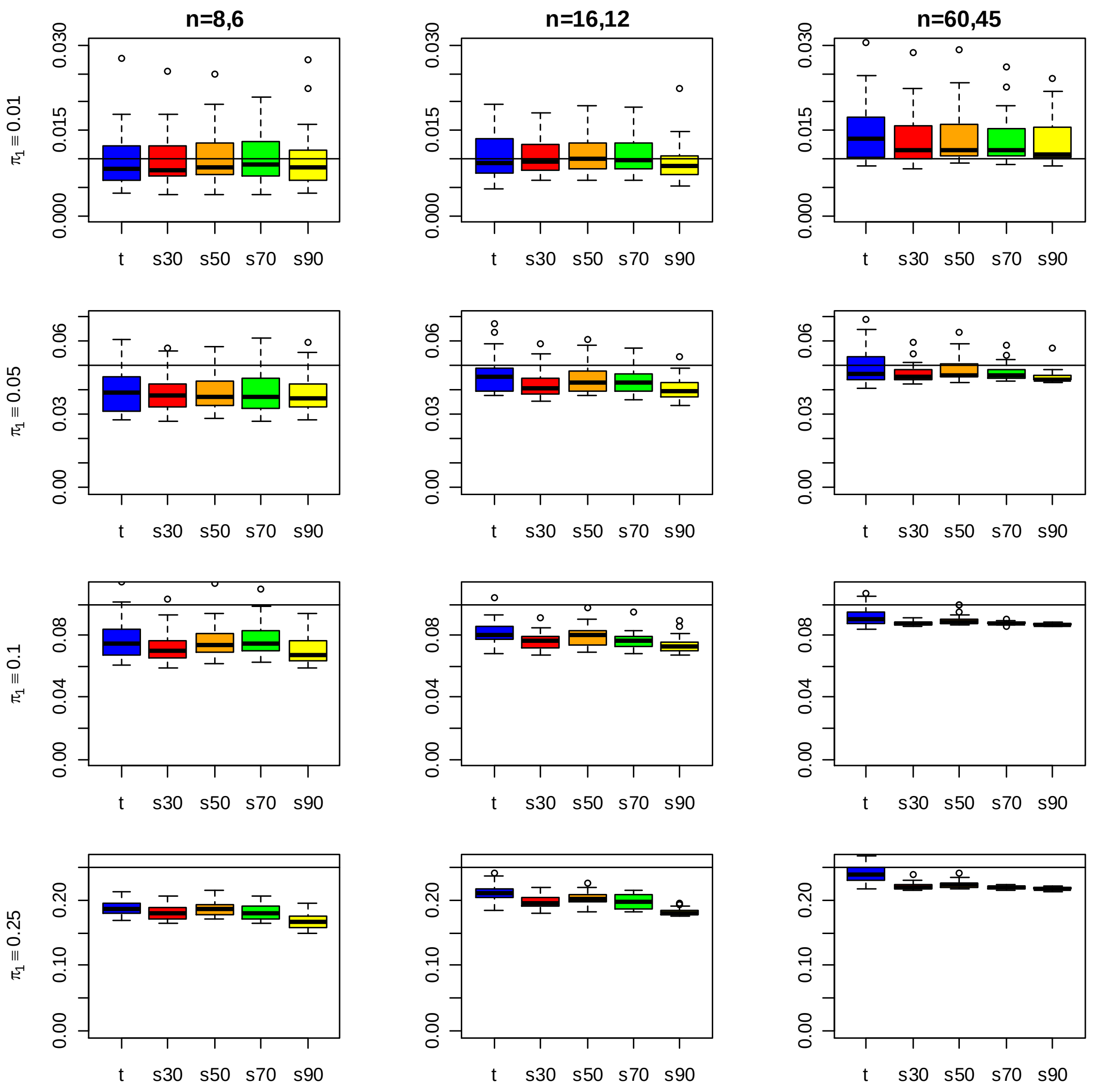

Figure 16. 'convest' estimates of π_1 for the t-statistic and four s-statistics in a type EV simulation. A reference line is drawn at π_1, the true proportion of differentially expressed genes.

APPENDIX B: RESULTS FOR UV1 SIMULATIONS

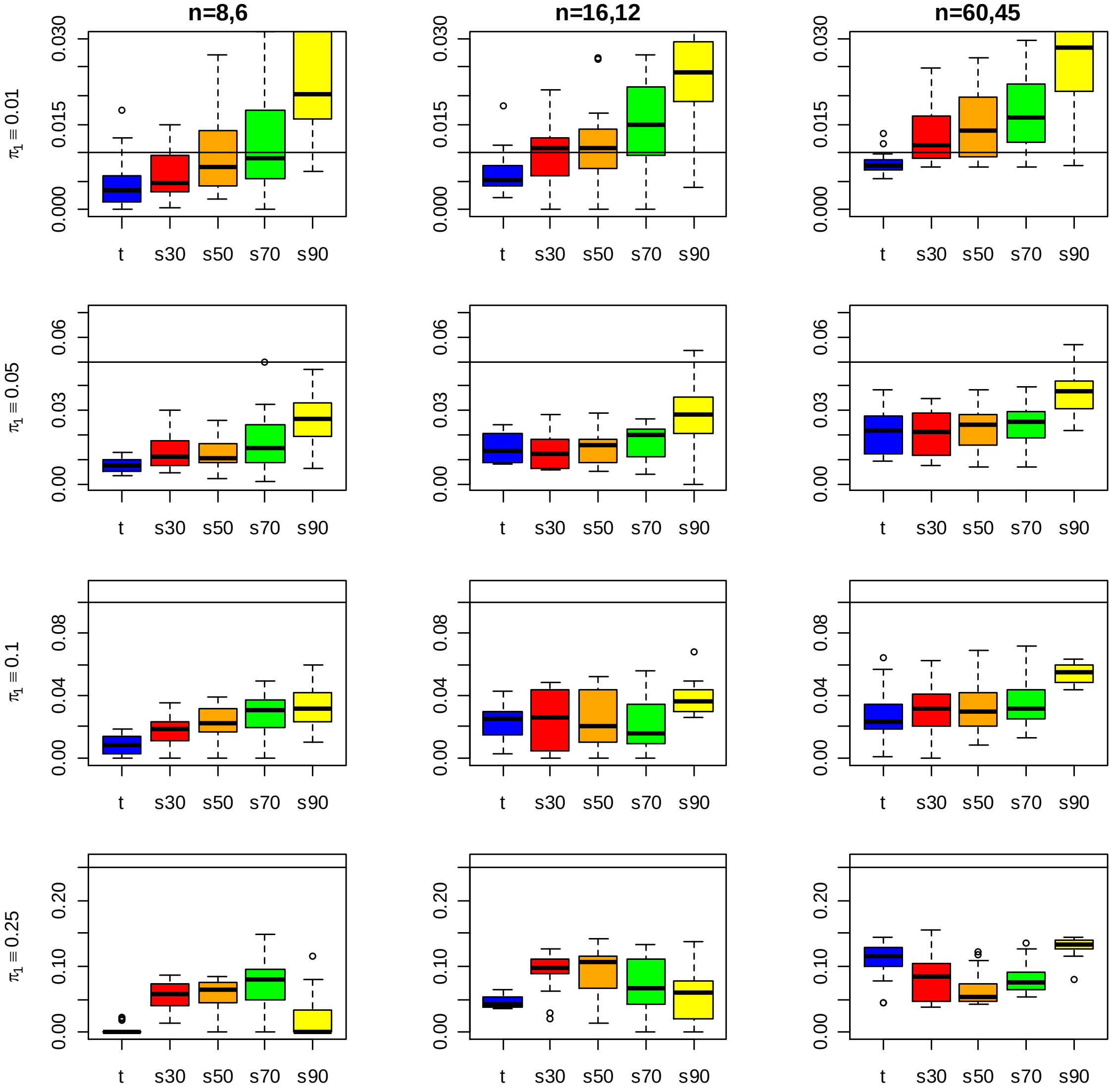

Figure 17. 'fdrtool' estimates of π$_1$ for the t-statistic and four s-statistics in a type UV1 simulation with the IQR rescaling factor and t-statistic input specification. A reference line is drawn at π$_1$, the true proportion of differentially expressed genes.

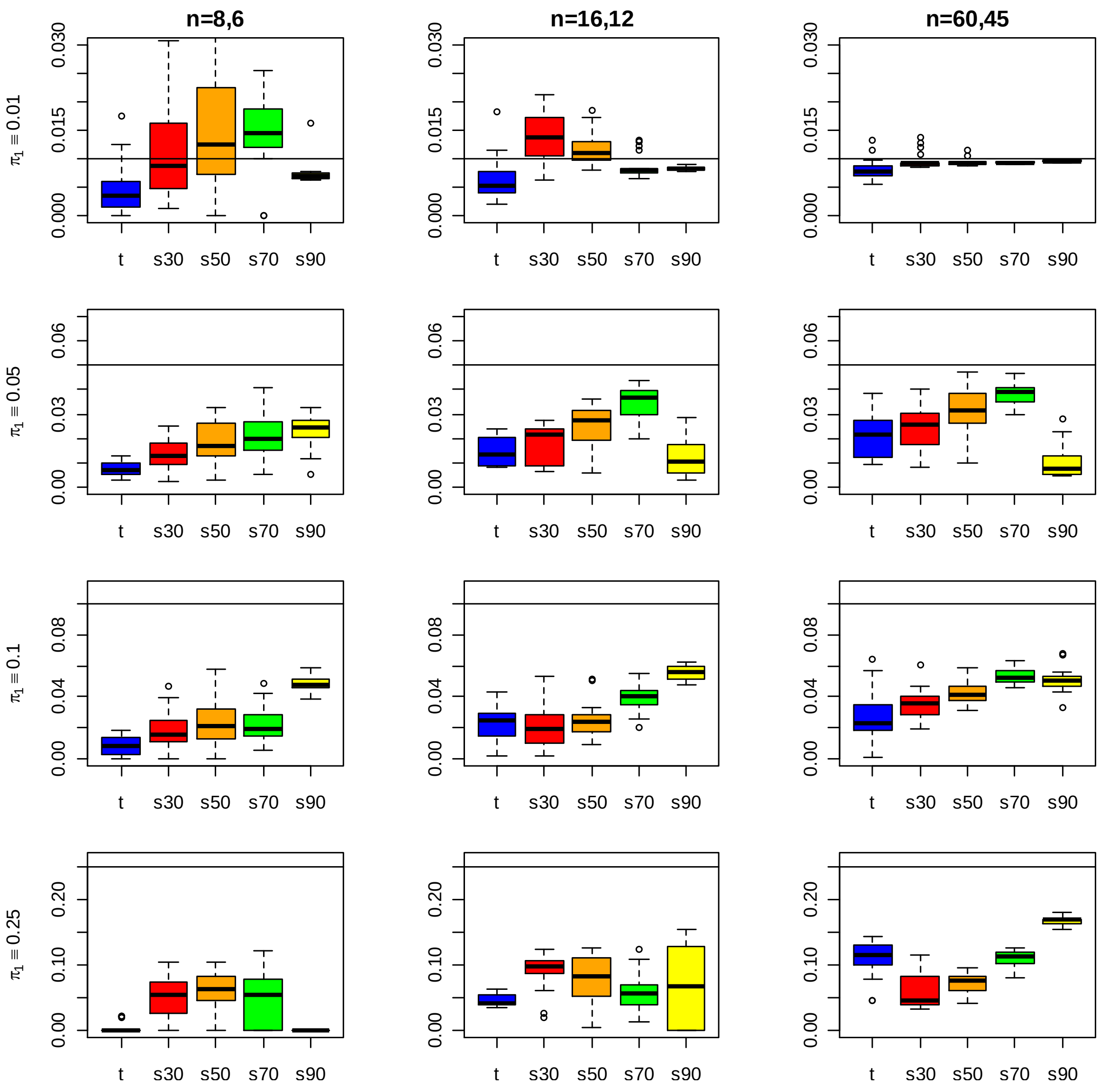

Figure 18. ‘fdrtool’ estimates of π_1 for the t-statistic and four s-statistics in a type UV1 simulation with the SD rescaling factor and t-statistic input specification. A reference line is drawn at π_1, the true proportion of differentially expressed genes.

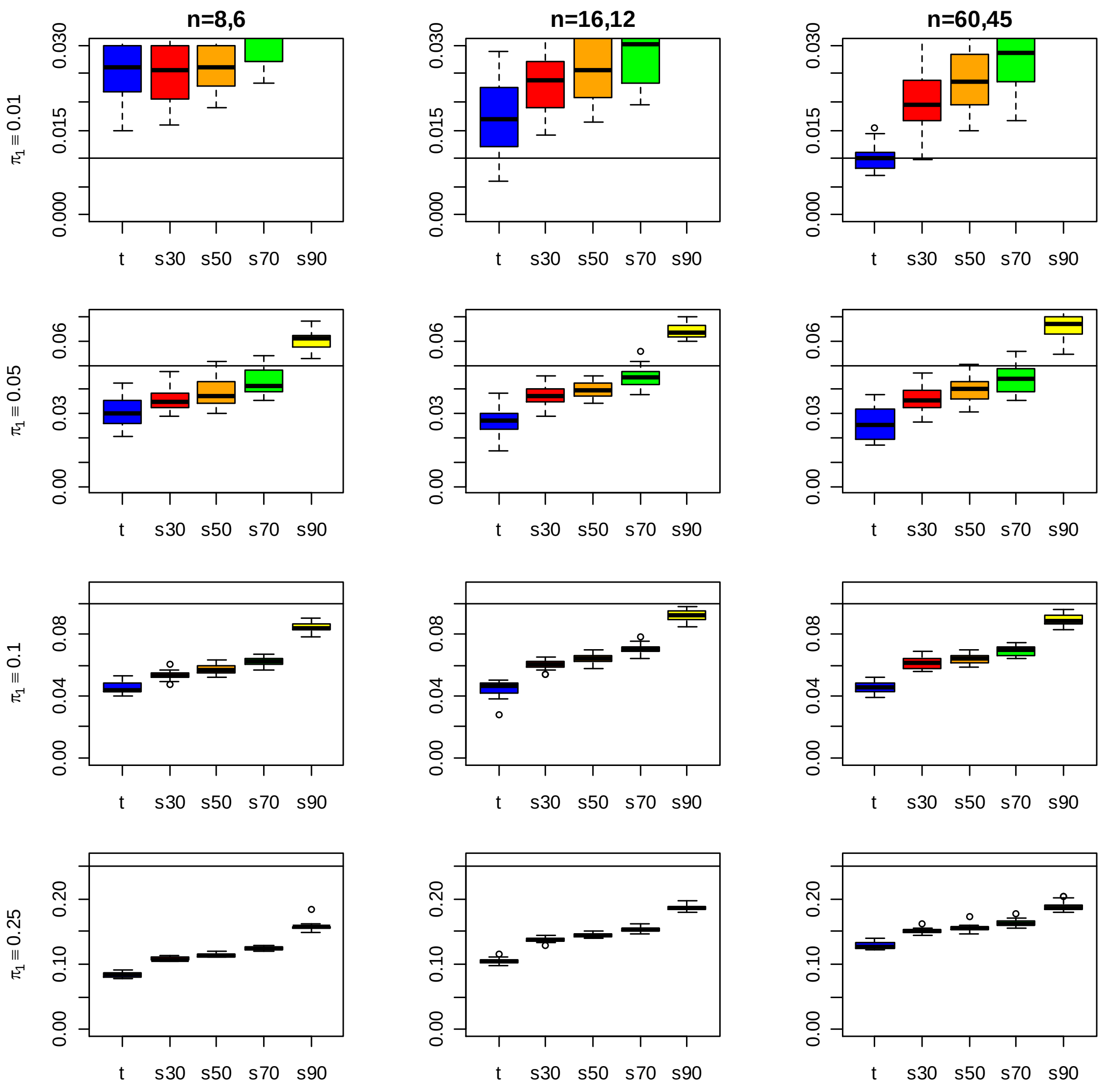

Figure 19. 'fdrtool' estimates of π_1 for the t-statistic and four s-statistics in a type UV1 simulation with the IQR rescaling factor and z-statistic input specification. A reference line is drawn at π_1, the true proportion of differentially expressed genes.

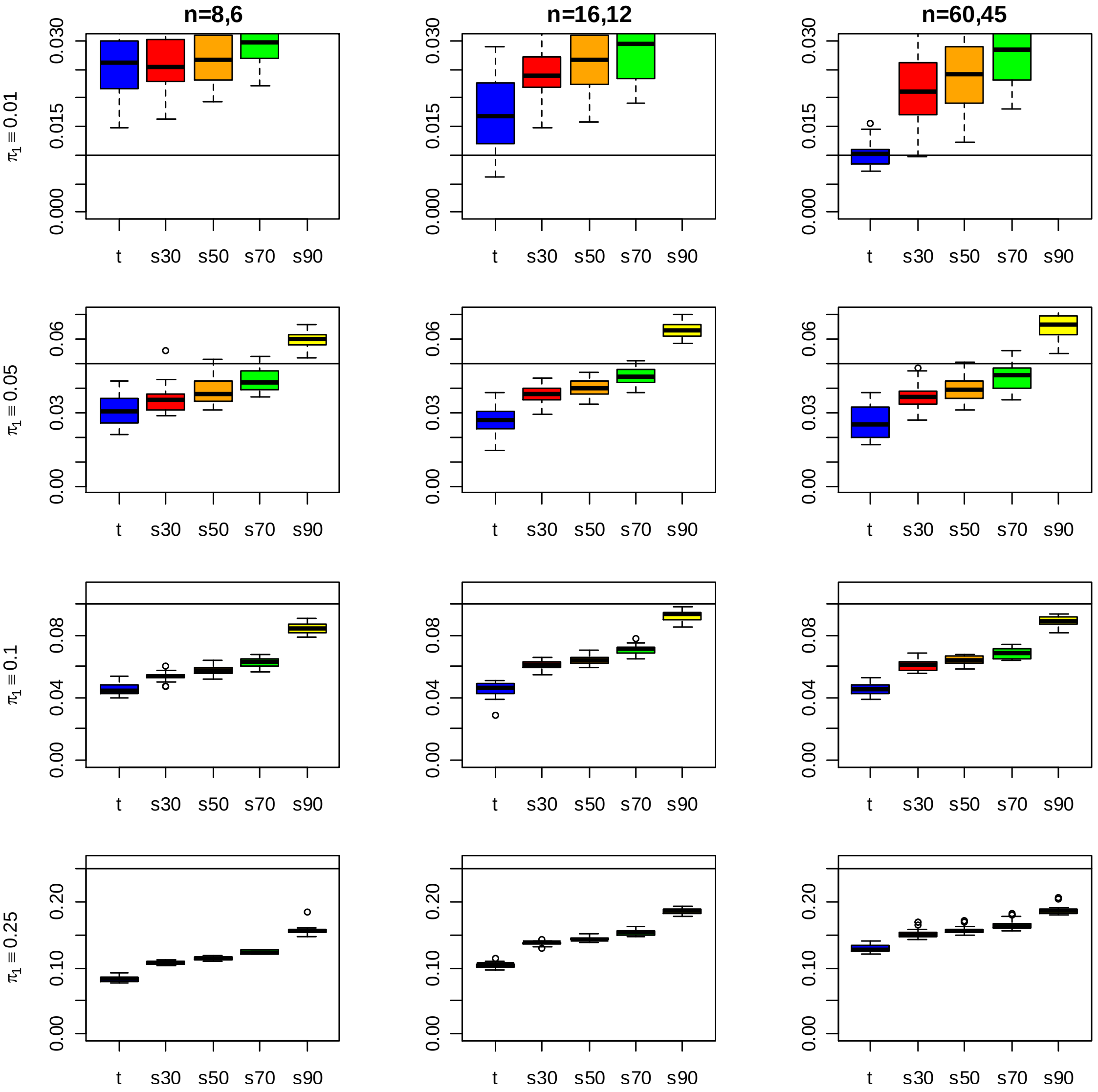

Figure 20. 'fdrtool' estimates of π_1 for the t-statistic and four s-statistics in a type UV1 simulation with the SD rescaling factor and z-statistic input specification. A reference line is drawn at π_1, the true proportion of differentially expressed genes.

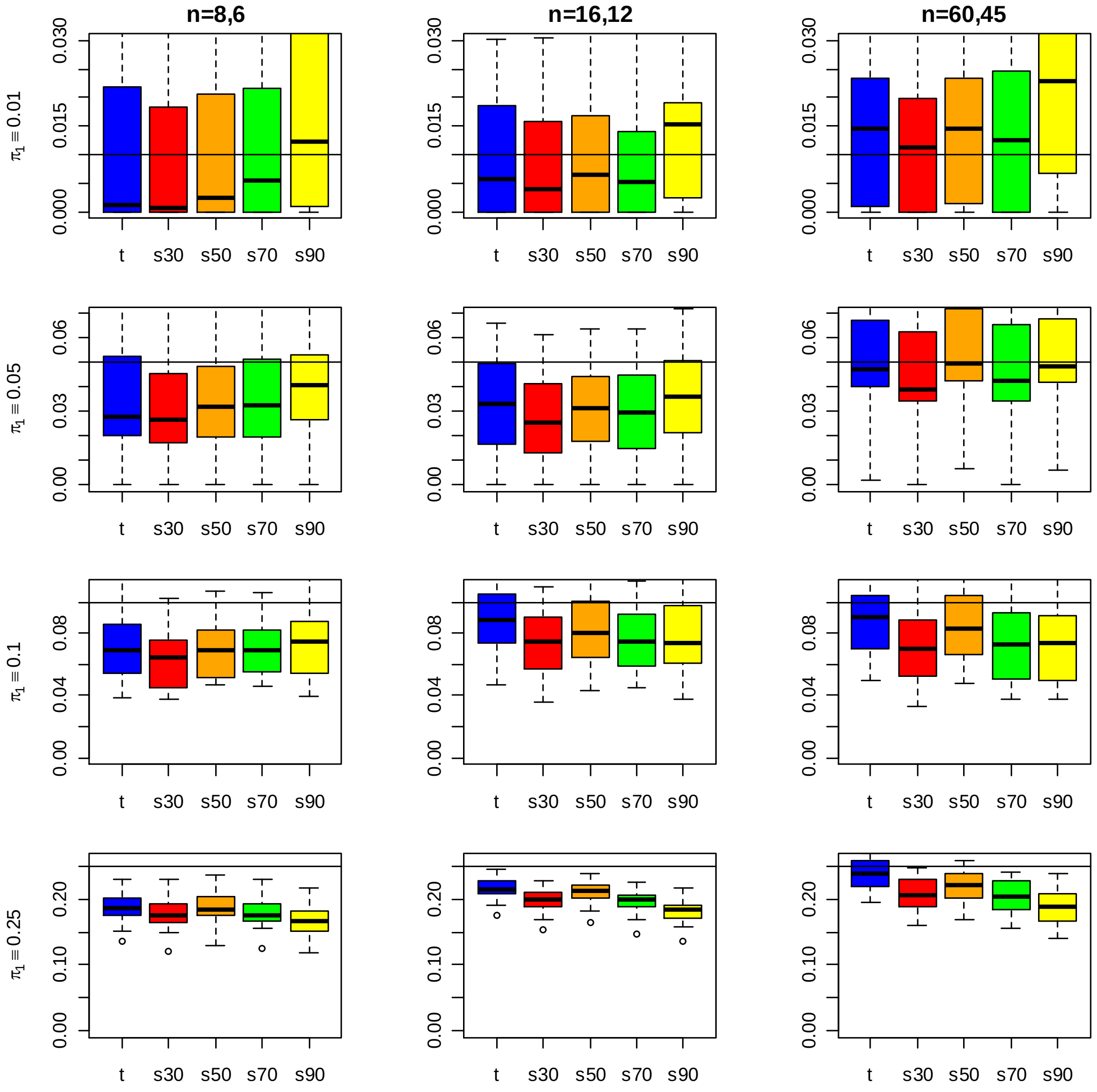

Figure 21. 'qvalue' estimates of π_1 for the t-statistic and four s-statistics in a type UV1 simulation. A reference line is drawn at π_1, the true proportion of differentially expressed genes.

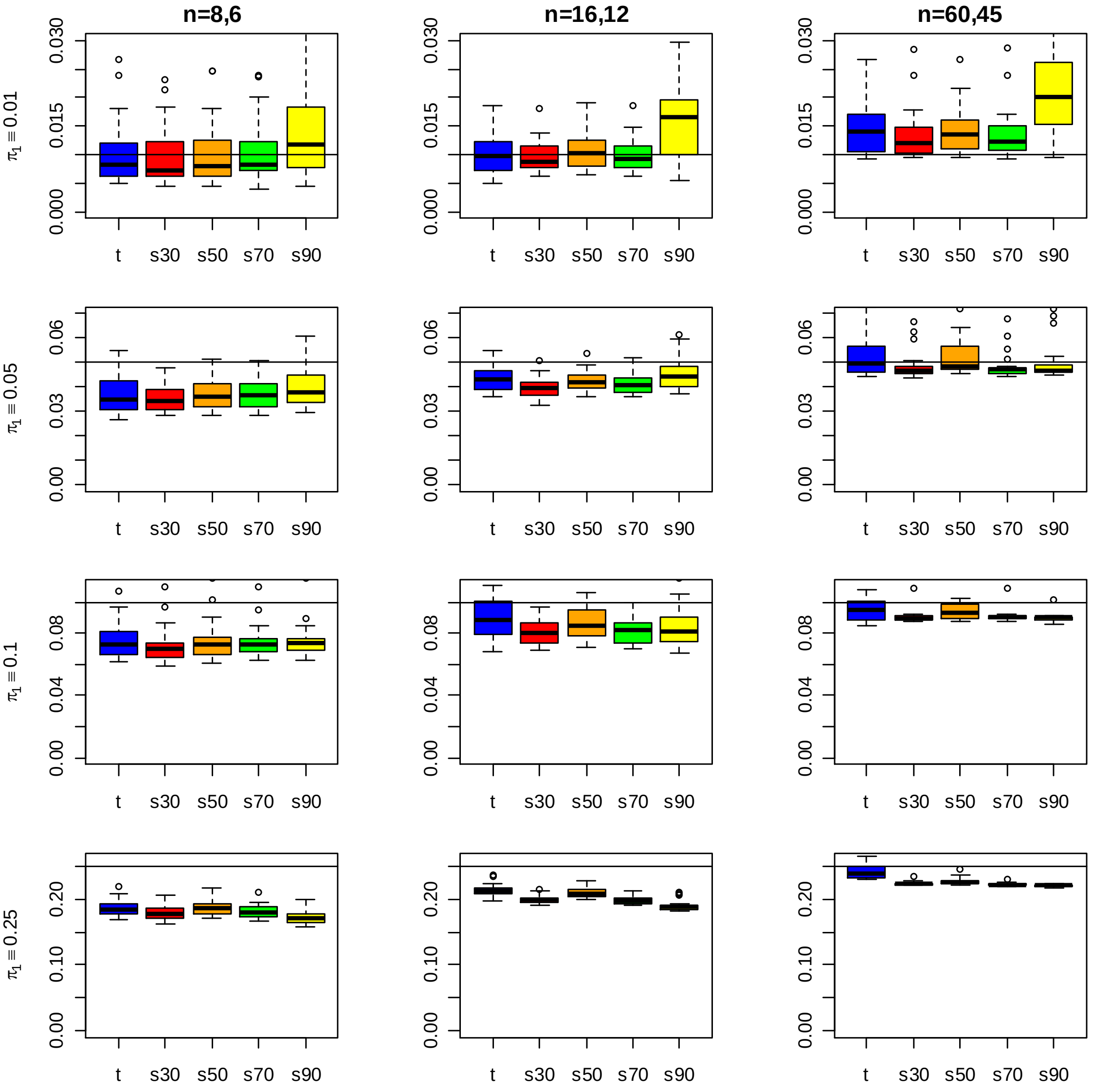

Figure 22. 'convest' estimates of π_1 for the t-statistic and four s-statistics in a type UV1 simulation. A reference line is drawn at π_1, the true proportion of differentially expressed genes.

APPENDIX C: RESULTS FOR UV2 SIMULATIONS

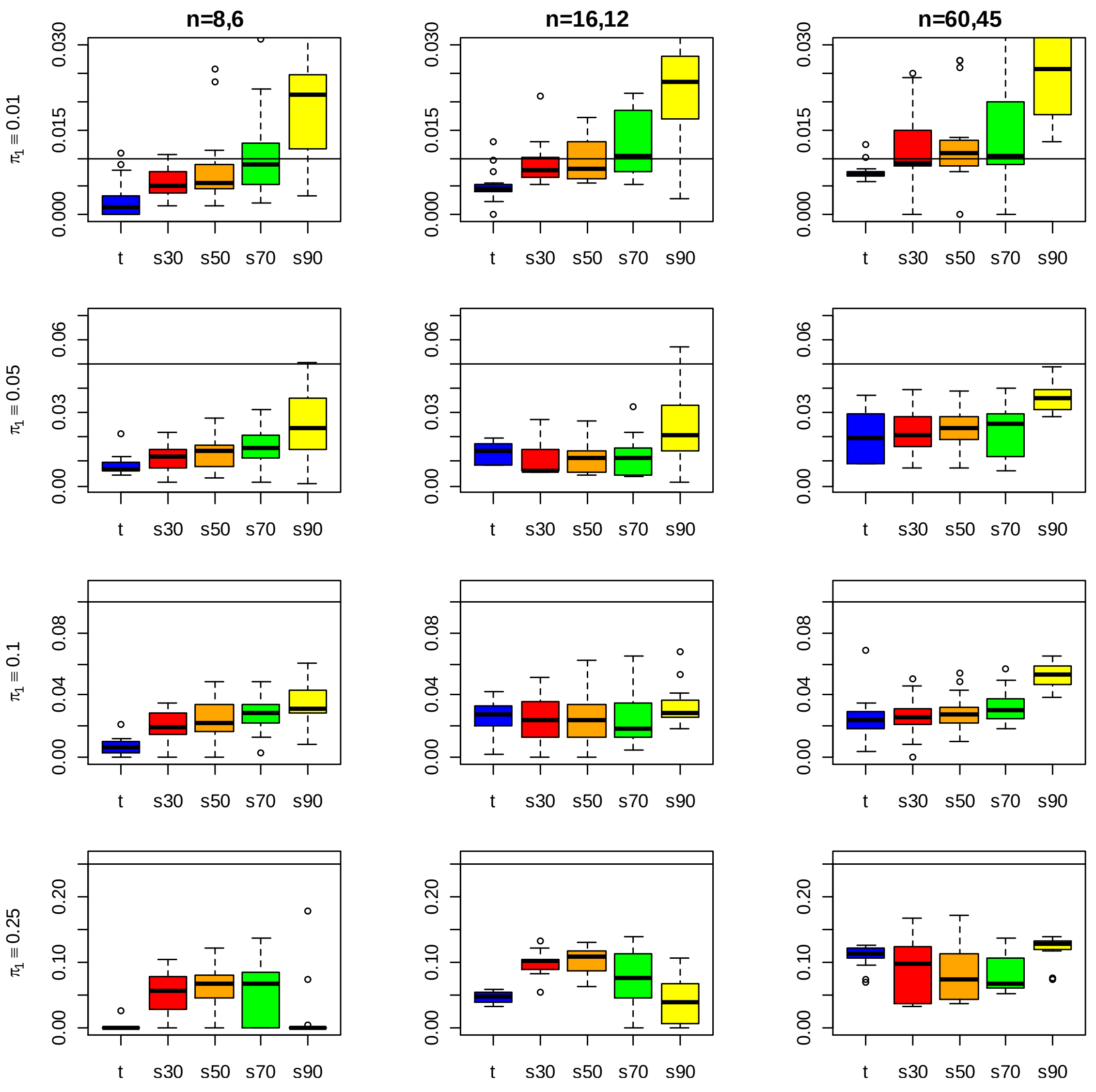

Figure 23. ‘fdrtool’ estimates of π_1 for the t-statistic and four s-statistics in a type UV2 simulation with the IQR rescaling factor and t-statistic input specification. A reference line is drawn at π_1, the true proportion of differentially expressed genes.

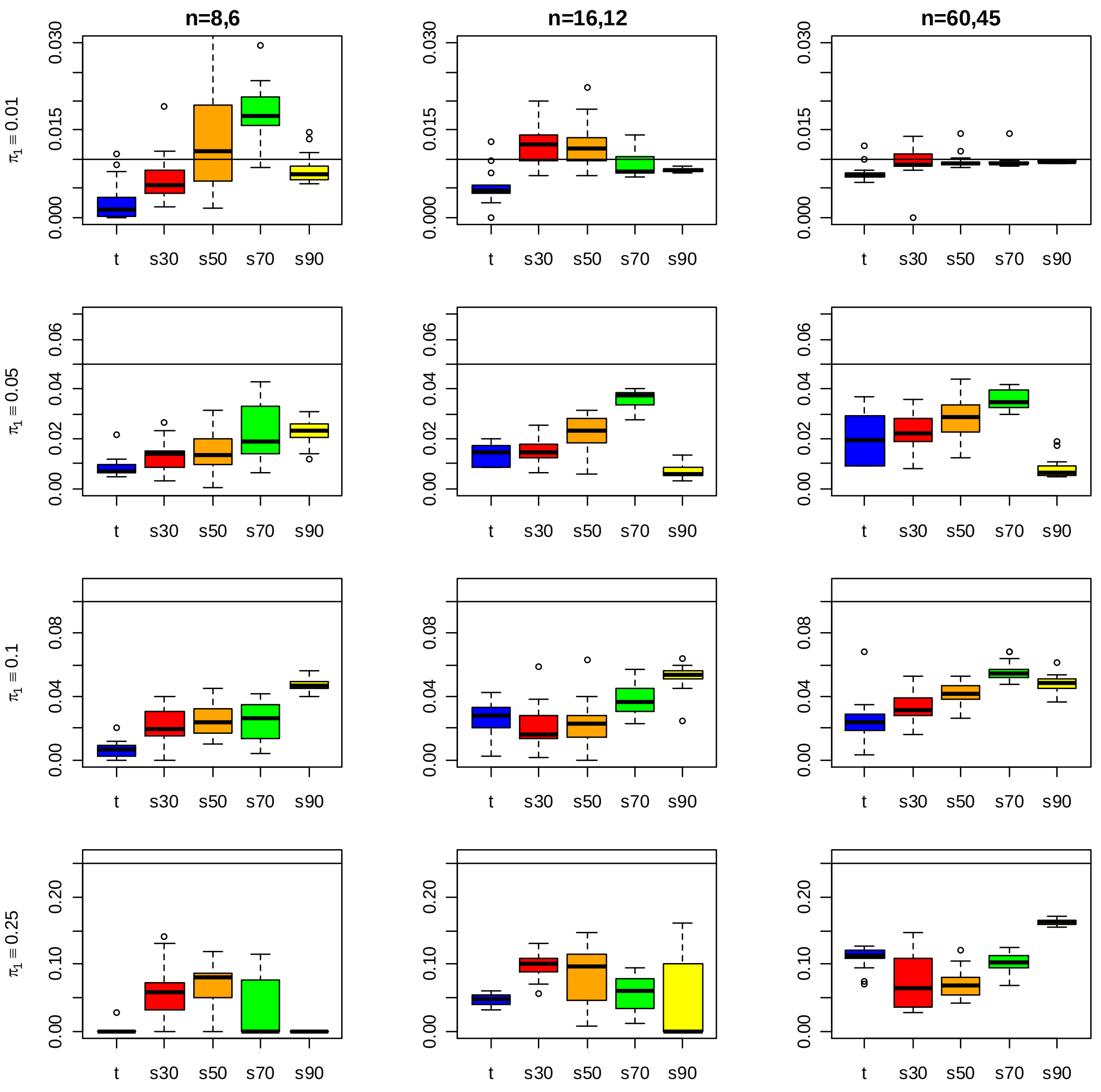

Figure 24. ‘fdrtool’ estimates of π_1 for the t-statistic and four s-statistics in a type UV2 simulation with the SD rescaling factor and t-statistic input specification. A reference line is drawn at π_1, the true proportion of differentially expressed genes.

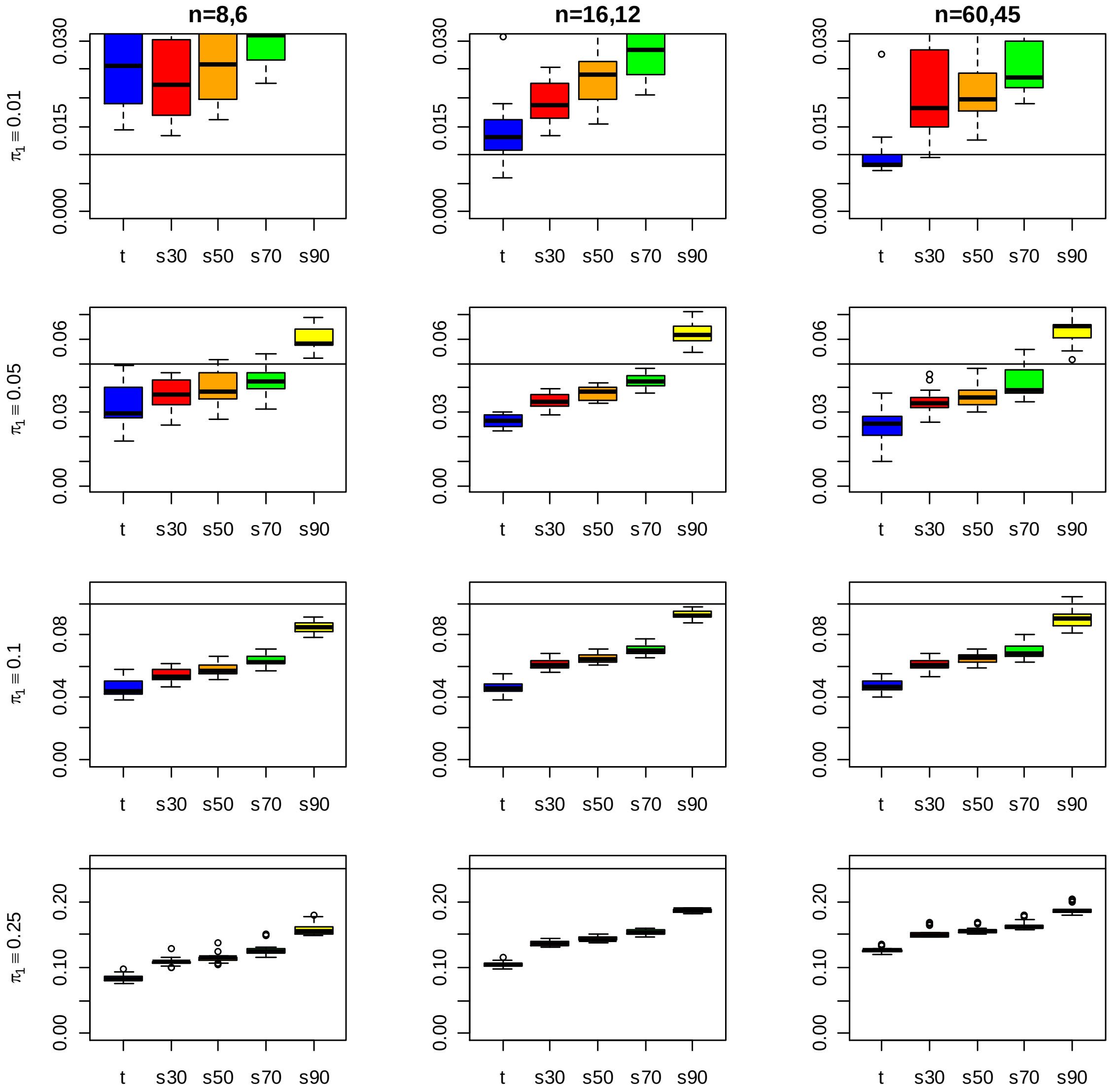

Figure 25. ‘fdrtool’ estimates of π_1 for the t-statistic and four s-statistics in a type UV2 simulation with the IQR rescaling factor and z-statistic input specification. A reference line is drawn at π_1, the true proportion of differentially expressed genes.

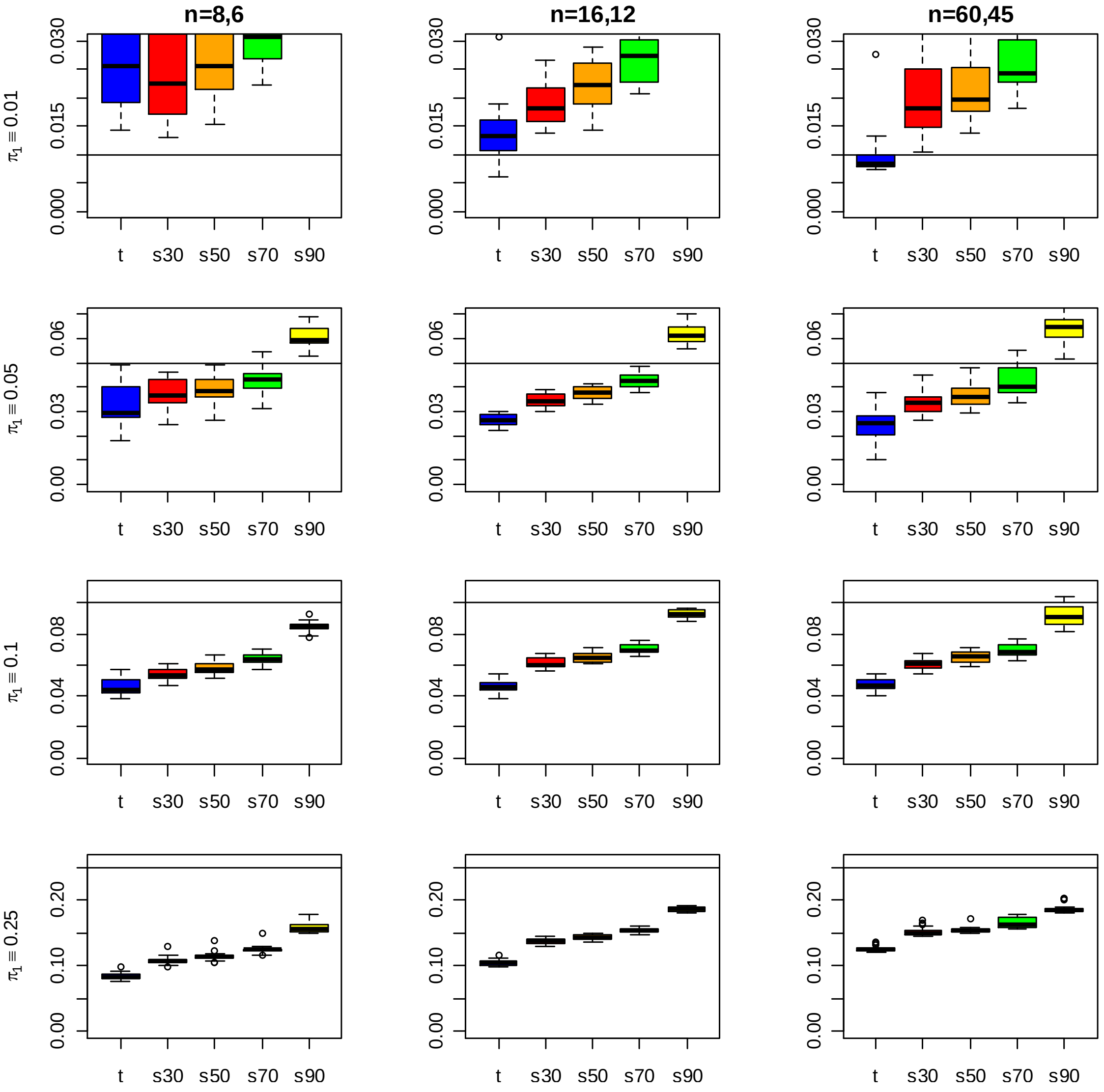

Figure 26. 'fdrtool' estimates of π_1 for the t-statistic and four s-statistics in a type UV2 simulation with the SD rescaling factor and z-statistic input specification. A reference line is drawn at π_1, the true proportion of differentially expressed genes.

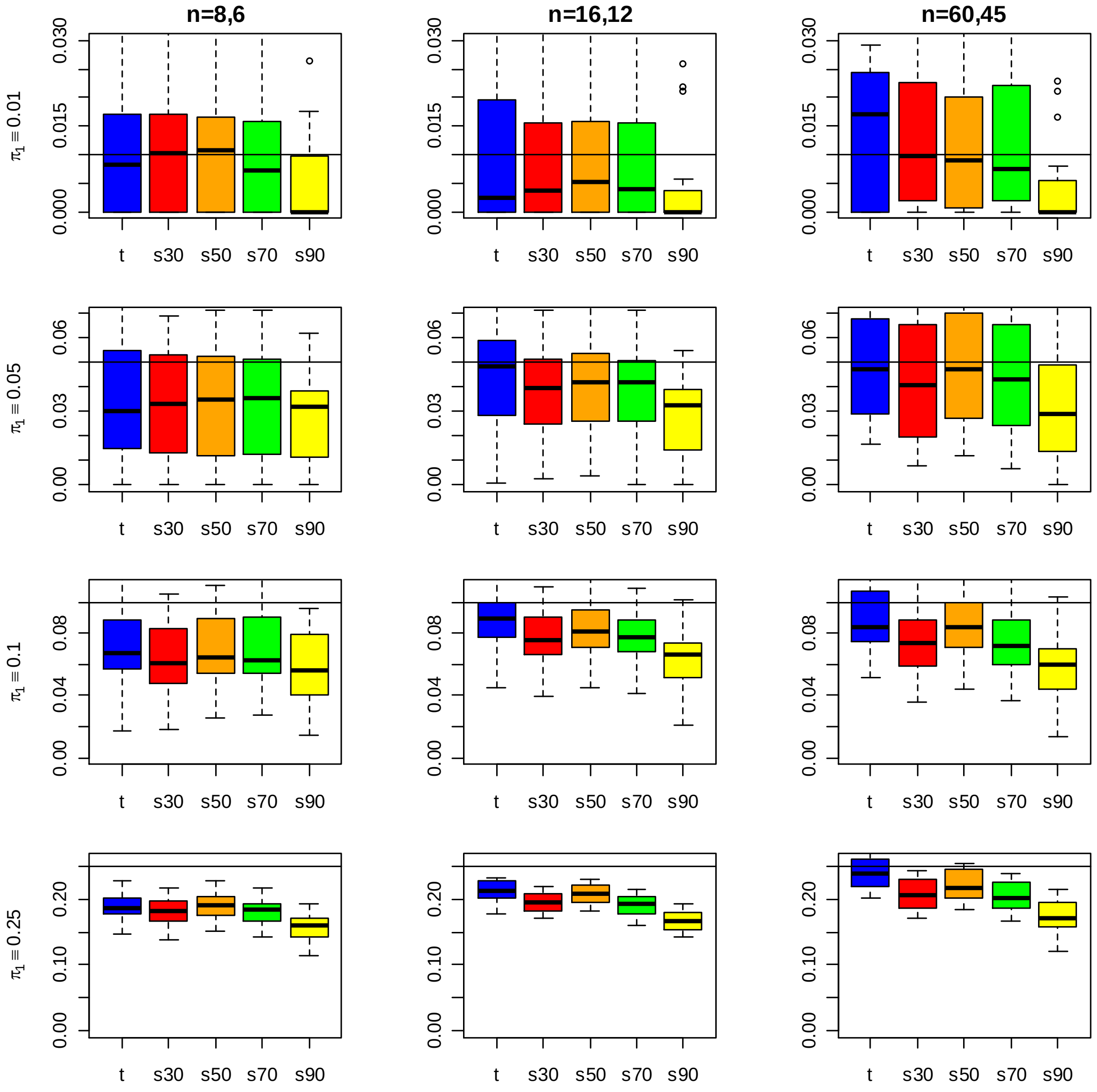

Figure 27. ‘qvalue’ estimates of π_1 for the t-statistic and four s-statistics in a type UV2 simulation. A reference line is drawn at π_1, the true proportion of differentially expressed genes.

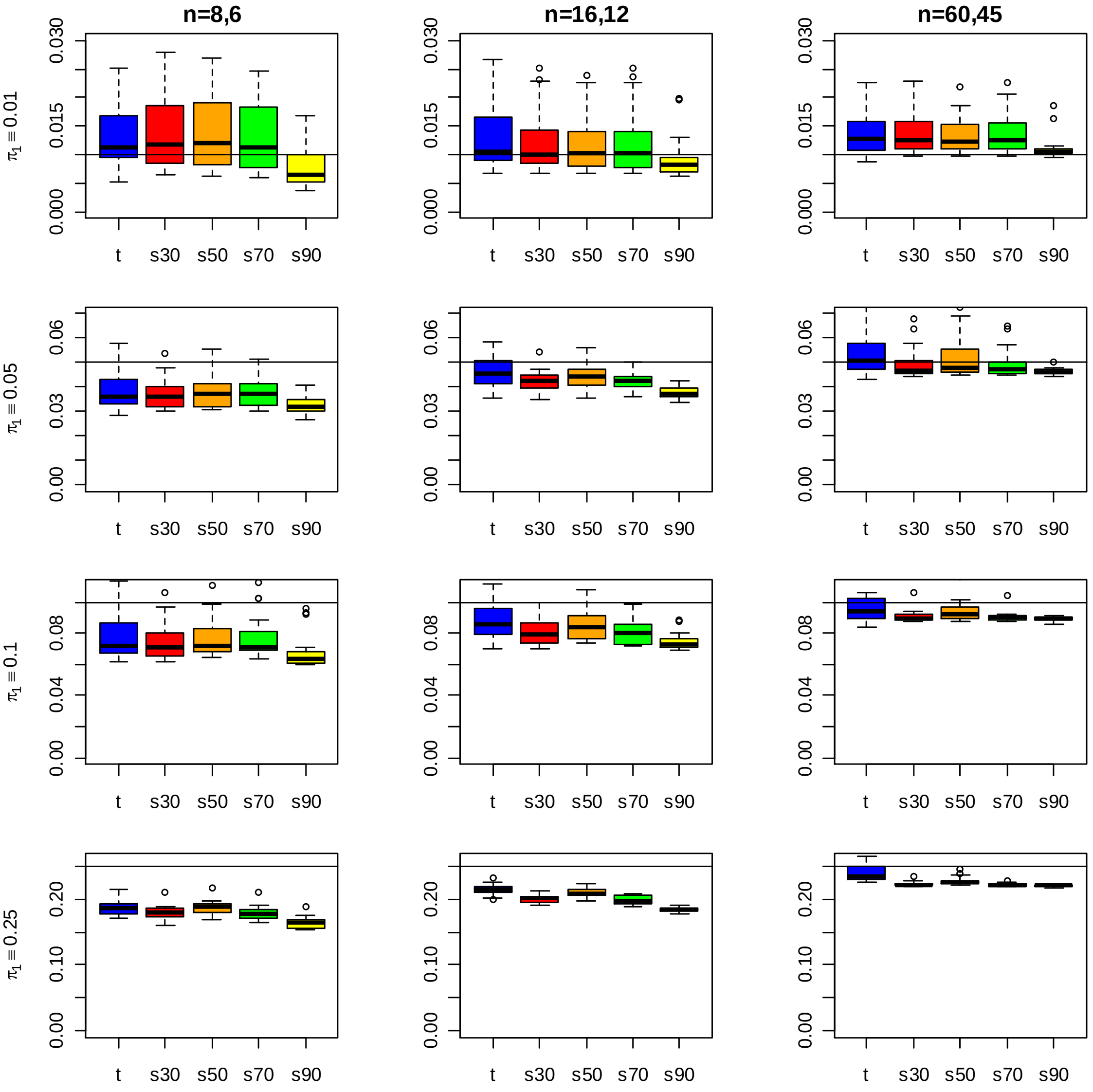

Figure 28. 'convest' estimates of π_1 for the t-statistic and four s-statistics in a type UV2 simulation. A reference line is drawn at π_1, the true proportion of differentially expressed genes.
